# Functional diversity and evolutionary strategies in nematophagous fungi and plant parasitic nematodes interactions - a review

**DOI:** 10.3389/fpls.2026.1938547

**Published:** 2026-09-08

**Authors:** Aurelio Ciancio, Ugo Picciotti

**Affiliations:** 1 Institute for Sustainable Plant Protection, National Research Council, Bari, Italy; 2 Department of Soil, Plant and Food Sciences, University of Bari Aldo Moro, Bari, Italy

**Keywords:** biological control, functional diversity, nematode-trapping fungi, secondary metabolites, soil ecology

## Abstract

Plant-Parasitic Nematodes (PPNs) cause substantial economic losses globally and severely undermine food security. In this context, nematophagous fungi (NFs) have emerged as promising agents of sustainable biocontrol, owing to their ability to attack, parasitise, and digest nematodes through diverse mechanisms. This review analyses the taxonomic diversity, mechanisms of action, and evolutionary relationships of NFs, highlighting the primary functional groups recognized for their ecosystem services in soil. These include trapping fungi, endoparasites, egg and female parasites, toxin producers, and species possessing mechanical attachment structures. Furthermore, the ecological and biological factors influencing the efficacy of these microorganisms are discussed, including the role of secondary metabolites, interactions with symbiotic bacteria, and the impact of soil micro-environmental conditions. Published phylogenetic frameworks suggest that capture devices represent pivotal traits in the evolutionary history of these organisms, pointing to distinct evolutionary pathways between adhesive and non-adhesive strategies. Overall, the biodiversity of NFs within soil microbial communities constitutes a strategic resource for developing innovative approaches to the sustainable management of PPNs.

## 1 Generalities and mechanisms of action

Nematodes are ubiquitous invertebrates comprising over 28,000 recognized species with diverse feeding mechanisms, encompassing pests of both plants and animals. They play a fundamental role within soil ecosystems (Rahman et al., 2023; Villarreal-Pérez et al., 2025). Plant-parasitic nematodes (PPNs) pose a severe threat to global food security, causing direct and indirect damages to crops. Global financial losses are estimated at approx. 157 billion USD annually, corresponding to a yield reduction of approx. 12.6% (Meel and Saharan, 2025). The management of PPNs remains highly challenging, particularly within organic farming systems. In recent years, various sustainable approaches using biocontrol agents have been investigated to provide effective alternatives to conventional management, which predominantly relies on synthetic nematicides or, when available, resistant crop varieties. Sustainable agroecosystem management necessitates the preservation of soil biodiversity and ecological functionality for future generations, an objective to which natural antagonists can significantly contribute (Shukuru et al., 2024).

The term “nematophagous fungi” (NFs) encompasses a diverse group of species capable of predating, attacking, or parasitising, and ultimately killing nematodes at various life stages to acquire nutrients. NFs are ubiquitous across nearly all environments, including tropical, forest, aquatic, and Antarctic ecosystems (Gray and Lewis Smith, 1984; Gray and Bailey, 1985; Swe et al., 2009; Tarigan et al., 2020; Sánchez-Martínez et al., 2023). They comprise approx. 700 species spanning diverse taxonomic groups, such as Ascomycota, Basidiomycota, Blastocladiomycota, and Zoopagomycota (de Freitas Soares et al., 2018; Khan et al., 2023; Villarreal-Pérez et al., 2025). Many of these species are polyphagous; in addition to attacking nematodes, they can colonise and parasitise other organisms, including non-nematode invertebrates (e.g. molluscs), plants, and other fungi. While some NFs are obligate parasites of nematodes, the majority are facultative saprotrophs capable of being cultured *in vitro*. Given their capacity to target both plant- and animal-parasitic nematodes, these fungi possess substantial potential for industrial exploitation as biocontrol agents (Lopez-Llorca et al., 2008; de Freitas Soares et al., 2018).

Given their widespread availability, ease of isolation, and survival in organic manure and composts, several NF species were initially proposed, and eventually tested, for the biological control of infective larvae of animal-parasitic nematodes (Wolstrup et al., 1996). However, given the specific scope of this review and the distinct ecology of such applications, this topic is not addressed here, as it warrants a dedicated discussion in a separate paper.

Hyphomycetes such as *Pochonia chlamydosporia*, *Purpureocillium lilacinum*, *Arthrobotrys* spp. or *Trichoderma* spp. have been extensively evaluated in biocontrol assays against major PPNs, predominantly root-knot and cyst-forming species (Kerry, 2000; Lopez-Llorca et al., 2008). Screening available isolates is widely regarded as essential for achieving acceptable efficacy, owing to intraspecific variations in key traits such as host specificity and virulence (Kerry, 2000). This isolate selection process further underscores the practical necessity of evaluating fungal diversity and biology within the specific environmental context of their intended application. Furthermore, certain isolate traits can be influenced by hyperparasites, including mycoviruses. Mycoviruses are present not only in phytopathogenic fungi such as *Cryphonectria parasitica* (Chun et al., 2020; Krstin et al., 2020) but also in NFs like *P. chlamydosporia* (Monteiro et al., 2016) and entomopathogens such as *Metarhizium anisopliae* (Leal et al., 1994).

Interactions between NFs and other microorganisms inhabiting soil or rhizosphere microcosms may significantly influence biocontrol efficacy, a phenomenon frequently observed when comparing results from controlled bioassays with field applications. Indeed, the poor transition from controlled laboratory conditions to field applications is often encountered deploying NF isolates, primarily due to unsuitable formulations and/or reduced fungus persistence. The inhibition and restricted development of fungal propagules introduced into soil is a well-documented phenomenon termed “soil fungistasis” (Mankau, 1962). For instance, field application of the endoparasites *Hirsutella rhossiliensis* to control the cyst nematode *Heterodera schachtii* in California failed due to fungivorous mites feeding on the fungal granules (Jaffee et al., 1996).

NFs include species exhibiting highly diverse phenotypic traits resulting from evolutionary adaptative radiation. Indeed, these fungi did not originate from a single, monophyletic radiation; rather, they comprise a wide diversity of taxa that have convergently evolved similar functional feeding strategies targeted on nematodes. Many of these organisms are hyphomycetes that produce specialized structures, often induced by the presence of their host, to capture and digest live nematodes (Villarreal-Pérez et al., 2025). Based on their mode of action, NFs are broadly categorized into five functional groups (**Table 1**): (I) trapping fungi (formerly referred to as predatory species), (II) nematode endoparasites, (III) egg and female parasites, (IV) toxin producers (Jansson and Lopez-Llorca, 2001), and (V) fungi forming specialized mechanical attachment or attack devices (Jansson and Lopez-Llorca, 2001; Lopez-Nuñez et al., 2022).

**Table 1:** Summary of NFs functional groups showing representative species, their trophic strategies, and most common target stages.

Functional group	Representative species	Trophic strategies	Target stage
Group I - Trapping fungi	*Arthrobotrys oligospora;* *Drechslerella dactyloides:* *Dactylella ellipsospora*	Adhesive networks; Constricting rings; Adhesive knobs	Juveniles and vermiform stages
Group II - Endoparasites	*Hirsutella rhossiliensis*, *Drechmeria coniospora*	Adhesive conidia, germinating and penetrating the host	Juveniles and adult vermiform stages
Group III - Egg/female parasites	*Pochonia chlamydosporia*, *Purpureocillium lilacinum*, *Lecanicillium saksenae*	Cuticle lysis followed by hyphal penetration with austoria	Eggs and adult females, egg masses
Group IV - Toxin producers	*Pleurotus ostreatus*, *Nematoctonus* spp.	Exude paralytic toxins and extracellular proteases	Juveniles and adult vermiform stages
Group V - Attacking through specialized mechanical devices	*Conocybe comatus*, *Haptoglossa* spp.	Forms spiny balls damaging the cuticle, followed by extruded toxins; gun cells injecting an infective sporoplasm	Juveniles and adult vermiform stages

### 1.1 Group I: trapping fungi

**Figure 1:**
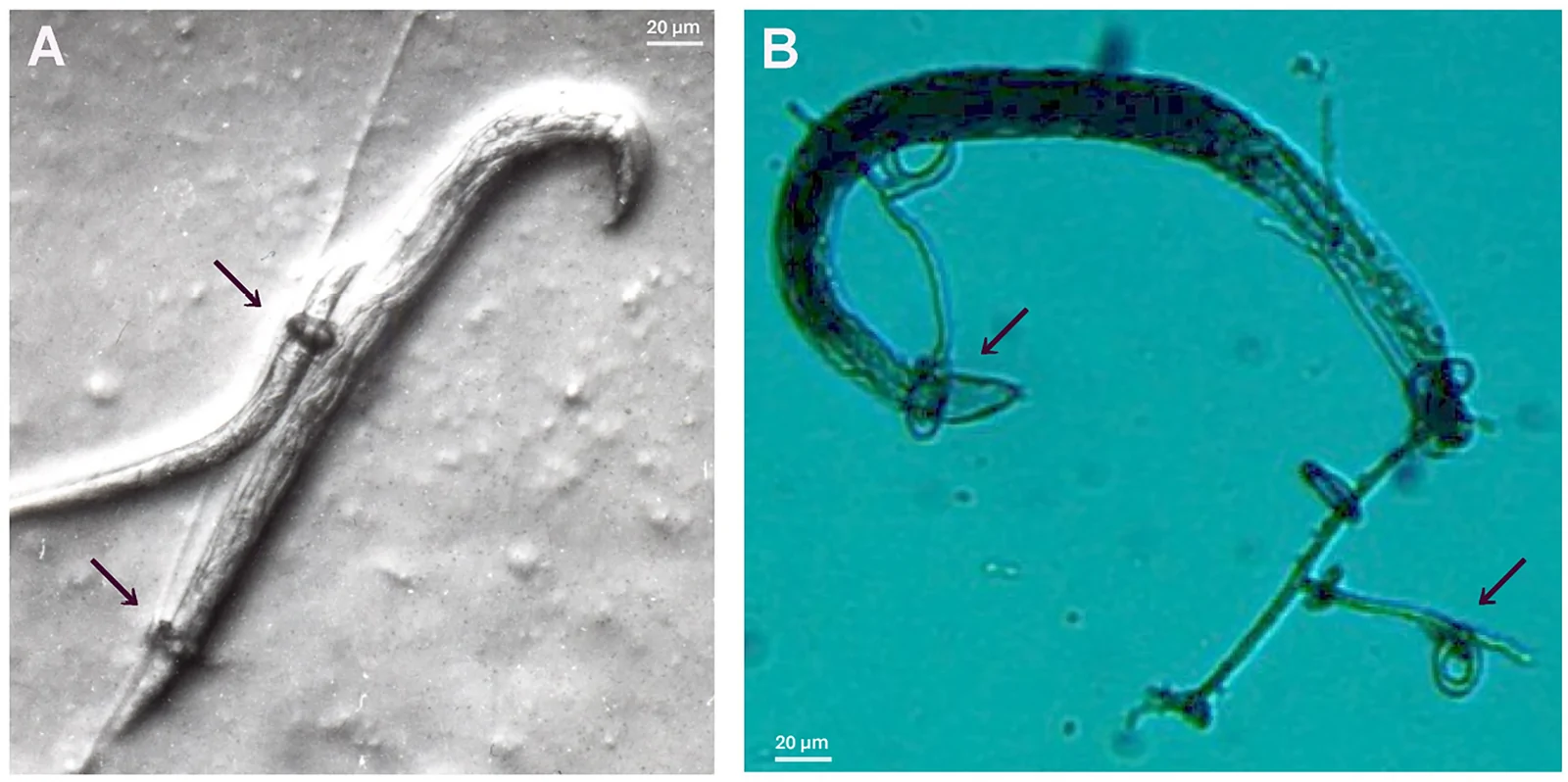
Nematode trapping fungi can be isolated from their preys, raised on 1.5% water agar. **(A)**. After adding a few g of soil or roots to the agar, free living microbiovorous nematodes start to colonize the substrate, followed by the emergence of NF fungi that capture them. The isolates can be easily obtained by collecting spores from the emerging conidiophores and spreading them on nutritive substrates such as PDA or CMA. NF isolates can be also obtained from captured specimens found during routine nematode counting. These specimens can be mounted on temporary slides in water for examination or placed on a nutritive substrate to cultivate the fungus **(B)**. In both cases shown, the traps consist of constrictor rings such as those of *Drechslerella dactyloides* (black arrows).

Trapping fungi have been known for over a century and comprise species characterised by the development of specialized hyphal structures that serve as functional traps, capturing nematodes via constricting rings or adhesive networks (**Figure 1**). These fungi belong predominantly to the genera *Arthrobotrys*, *Dactylellina* and *Drechslerella.*

Trophic specialisation towards predation serves to acquire nutrients from alternative sources, which is necessary for competing with other rhizosphere microorganisms. As polyphagous, facultative saprotrophs, many NFs can feed on and recycle organic compounds present in their microenvironment. They are ubiquitous, easy to isolate, and propagate. The development of specialized structures is triggered to target nematodes, marking the transition from a saprotrophic to a predatory lifestyle (Zhang et al., 2025). Under nitrogen-limiting soil conditions, trapping fungi shift from saprotrophy to predation, forming adhesive hyphal traps that capture active nematodes upon contact. These traps are characterised by an extracellular matrix of fibrils containing carbohydrates and glycoproteins, which are pivotal for immobilising the prey (Wang et al., 2023). Several fungi producing such hyphal structures actively attract nematodes by exuding volatile organic compounds (VOCs) that mimic food sources or sexual signals (Wang et al., 2023). Representative examples include *Dactylellina cionopaga*, which forms adhesive branches, and *Arthrobotrys oligospora*, which produces adhesive networks that attract and entrap nematodes (Rahman et al., 2023).

Some NFs establish associations with endosymbiont bacteria that modulate trap formation. Endosymbiotic bacteria within fungi are known to influence host growth, reproduction, and key biological functions, frequently acting in synergy with their fungal host. For example, bacterial species such as *Bacillus licheniformis*, *Achromobacter xylosoxidans* and *Stenotrophomonas maltophilia* have been shown to markedly induce trap formation in NFs like *Arthrobotrys musiformis* (Zheng et al., 2024).

Other NFs are characterised by the production of adhesive hyphal branches i.e., *D. cionopaga*, or are equipped with adhesive knobs that stick to the host cuticle, thereby capturing nematodes i.e., *Dactylella ellipsospora*. Some of these species demonstrated to be effective against several PPNs with a potential for commercial bioformulations, including *D. ellipsospora* (formerly *Monacrosporum ellipsosporum*) producing adhesive knobs, or *Hirsutella rhossiliensis*, infecting nematodes through adhesive conidia (Gaspard and Mankau, 1987; Jaffee and Muldoon, 1995; Devi, 2018). However, some of these taxa exhibit slowly grow rates, a trait shared with *Hyalorbilia oviparasitica*, a hyphomycete capable of regulating *Heterodera schachtii* populations under field conditions and conferring long-term suppressiveness (Westphal and Becker, 2001; Olatinwo et al., 2006; Becker et al., 2013; Witte et al., 2020). *Drechslerella dactyloides* develops specialized constricting rings to capture nematodes; nevertheless, its capacity to promote fungal proliferation is limited by soil biotic and abiotic factors that inhibit spore germination (soil fungistasis). To overcome this restriction, *D. dactyloides* has evolved the ability to produce traps directly from conidia, called “conidial traps”. The induction of these structures has been linked to bacteria belonging to the genera *Acinetobacter* and *Pantoea*, likely representing an adaptive survival mechanism (Zhang et al., 2025).

Although trapping fungi have been investigated for over a century, novel species are still being discovered, further expanding the arsenal of potential biocontrol agents against PPNs. For instance, the fungus *Arthrobotrys mendozadegivensis* was recently isolated and described from decaying wood. This species is characterised by distinct morphological features compared to other *Arthrobotrys* spp., including smaller conidia and chlamydospores, alongside three-dimensional adhesive networks exhibiting nematicidal activity (Gutiérrez-Medina et al., 2024).

### 1.2 Group II: endoparasitic fungi

**Figure 2:**
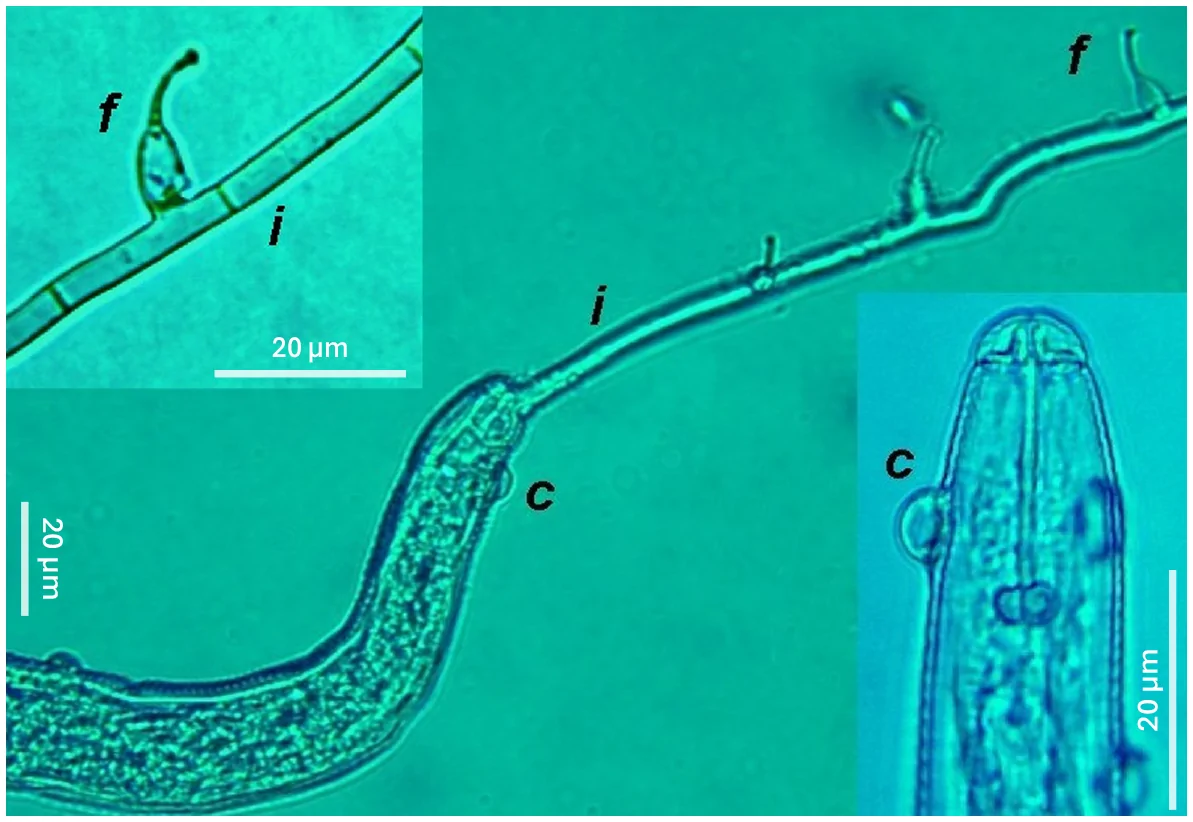
Hyphae of *Hirsutella rhossiliensis* emerging from a parasitised juvenile of the pea cyst nematode *Heterodera goettingiana*. The hyphae (i) produce phialides (f) on which adhesive conidia (c) develop that adhere to the host after a passive contact occurs in the soil microcosm. The conidia, germinating, penetrate the nematode body and develop an infectious mycelium that digests its contents. New hyphae then emerge, at the end of the propagation phase, from the remains of the nematode, thus completing the parasitic cycle.

The second functional group of NFs comprises endoparasitic species that infect nematodes primarily via adhesive conidia. These propagules, produced by conidiophores, adhere to the host cuticle upon contact. Most of these fungi are semi-obligate nematode parasites, completing their vegetative development preferentially inside the parasitised hosts (Lopez-Llorca et al., 2008). This group includes representative taxa such as *Hirsutella rhossiliensis*, *Drechmeria coniospora*, *Haptocillium* spp. (formerly *Verticillium*) and *Harposporium* spp. Following adhesion, the infectious conidia germinate to form an infection peg and internal trophic hyphae, which proliferate within and digests the internal contents of the host, frequently targeting motile juvenile stages. When complete the digestion of the host, the vegetative mycelium ruptures the cuticle and emerges from the cadaver, developing conidiophores to release new propagules (**Figure 2**).

### 1.3 Group III: egg and female parasites

The third functional group of NFs comprises endoparasites whose infectious hyphae differentiate appressoria to penetrate and attack adult females and the eggs. These fungi breach the eggshells to target the developing embryonic stages of nematodes, inducing cellular damage, arresting embryonic development, and increasing mortality in a dose-dependent manner (Arunachalam et al., 2025). Key species in this group include *P. chlamydosporia*, a widespread parasite of root-knot and cyst nematodes. During infection, *P*. *chlamydosporia* colonise eggs by producing nematicidal metabolites alongside a suite of hydrolytic enzymes (specifically chitinases, proteases, glucanases) that degrade chitin-rich matrix of the eggshell (Kerry, 2000; Lopez-Llorca et al., 2008, 2010; Picciotti et al., 2023). Furthermore, the secondary metabolites exuded by these fungi compromise embryonic physiology, reproductive fitness, and overall survival (Arunachalam et al., 2025).

Soil-inhabiting species, such as *Purpureocillium lilacinum*, are also included within this group. Egg and female parasites encompass additional taxa frequently encountered in the soil and rhizosphere microcosms, belonging to genera such as *Verticillium* (e.g. *V. leptobactrum*) and *Fusarium*. These taxa do not develop specialized hyphal structures for nematode adhesion or mechanical penetration and, moreover, include severe phytopathogenic species. Nevertheless, certain isolates have demonstrated the capacity to penetrate the eggs of cyst and root-knot PPNs, owing to high enzymatic activity (McLean and Lawrence, 1995; Viaene and Abawi, 1998; Gao et al., 2008; Regaieg et al., 2011; Manzanilla-López et al., 2013). The deployment of fungi within *Fusarium* and *Verticillium* as biocontrol agents remains controversial; despite the occurrence of isolates that act as beneficial endophytes for plants, many of them are closely related to, or even co-specific, with well-known plant pathogens. Indeed, *Fusarium* wilts are frequently exacerbated by nematode infection, which provides entry sites for fungal invasion (Morrell and Bloom, 1981; Okungbowa and Shittu, 2012).

*Lecanicillium saksenae* exhibits nematicidal efficacy through multifaceted mechanisms, including direct parasitism and destructive enzymatic activity. This fungus can penetrate the gelatinous matrix encasing *Meloidogyne incognita* egg masses, enabled by the secretion of proteolytic and chitinolytic enzymes. The structural breakdown of eggs and the subsequent inhibition of hatching rely on enzymes translocated into the eggshell via the appressorium; in particular, the synergistic action of proteases and chitinases effectively degrades the structural components of the eggshell (Sreeja et al., 2025).

### 1.4 Group IV: toxins producers

Toxin-producing NFs immobilise nematodes via secreted toxins prior to the hyphal penetration through the cuticle. The genus *Pleurotus* includes several toxin-producing species, most notably *Pleurotus ostreatus*. Biological control utilising edible mushrooms represents a sustainable alternative for the management of parasitic nematodes, with particular interest focussed on the proteases and metabolites produced by various *Pleurotus* spp. These fungi act by exuding paralytic toxins and extracellular proteases that immobilise the nematode and degrade its cuticle (Toledo da Silva et al., 2024). Furthermore, *Coprinus comatus* has been shown to exhibit similar nematicidal traits (Luo et al., 2004), suggesting that nematophagous capacity may be widespread among both edible and non-edible Basidiomycota. The earliest account of the efficacy of such thermostable toxins was reported by Giuma and Cooke (1972), who demonstrated the lethal effect of *Nematoctonus* spp. spores, following adhesion to juveniles of *Panagrellus redivivus* (Barron, 1977).

Numerous studies have investigated the antagonistic activity exerted by soil fungi against PPNs, highlighting both the role of various secondary metabolites and their complex interactions with other rhizosphere organisms (Kerry, 2000; Westphal and Becker, 2001; Lopez-Llorca et al., 2010; Manzanilla-López et al., 2013; Degenkolb and Vilcinskas, 2016; Meel and Saharan, 2025). For example, several *Aspergillus* species are highly prevalent in soil ecosystems and have been reported to suppress PPN populations (Shemshura et al., 2016; He et al., 2020). Furthermore, species such as *A. niger* and *A. terreus* produce a wide array of hydrolytic enzymes, particularly xylanases and cellulases, that contribute to nematophagous activity and plant growth promotion (Alhazmi et al., 2025). Nonetheless, *Aspergillus* is not broadly categorized as a true nematophagous genus. These fungi are among the most abundant fungi globally, exhibiting low selectivity regarding abiotic and biotic conditions, and several species are well-documented as plant pathogens (Garganese et al., 2016; Mincuzzi et al., 2023). A wide variety of aspergilli are also opportunistic human and animal pathogens (Krijgsheld et al., 2013). Moreover, *Aspergillus* spp. represent one of the main genera (alongside *Penicillium* spp. and *Alternaria* spp.) known to synthesize mycotoxins (Richard, 2007; Sanzani et al., 2019). This mycotoxigenic potential poses significant biosafety risks, precluding their commercial exploitation against PPNs despite their suppressive action (Krif et al., 2024; Zoubi et al., 2025).

### 1.5 Group V: fungi forming specialized mechanical attachment devices

Finally, the fifth functional group encompasses species that produce specialized mechanical structure designed to physically damage the nematode cuticles. Once cuticular integrity is compromised, the fungus differentiates a penetration peg or haustorium to invade the host tissues. This strategy is deployed by NFs such as the basidiomycetes *Conocybe apala* and *Stropharia rugosoannulata* (Lopez-Nuñez et al., 2022). Notably, certain NFs rely on a combination of attack mechanisms to entrap and kill their prey. An example is *C. comatus*, which forms distinctive “spiny balls” to inflict physical damage on the nematode cuticle while simultaneously exuding paralytic toxins to immobilise and kill the host. The synergistic action of these dual mechanical and chemical strategies markedly enhances the mortality and predatory efficiency (Luo et al., 2007).

The “gun cell” of the genus *Haptoglossa* (Lagenidiales, Oomycetes) represents another example of a highly sophisticated infection structure; it acts as a biological gun, firing an harpoon-like apparatus that breaches the host cuticle to inject an infective sporoplasm (Robb and Lee, 1986; Hakariya et al., 2002). A comparable attack mechanism has evolved in Microsporidia, a group of obligate intracellular parasites closely related to Fungi, which utilise an infectious filament coiled within the spores. Upon contact with the host, this filament is rapidly extruded, injecting the sporoplasm directly into the host. Microsporidia are unculturable microorganisms that lack canonical mitochondria, representing an early-diverging lineage of Fungi. Although many of microsporidians infect insects or molluscs, certain taxa have been documented to parasitise nematodes, including *Microsporidium rhabdophilum*. These taxa have been reported primarily as endoparasites of free-living nematodes.

**Figure 3:**
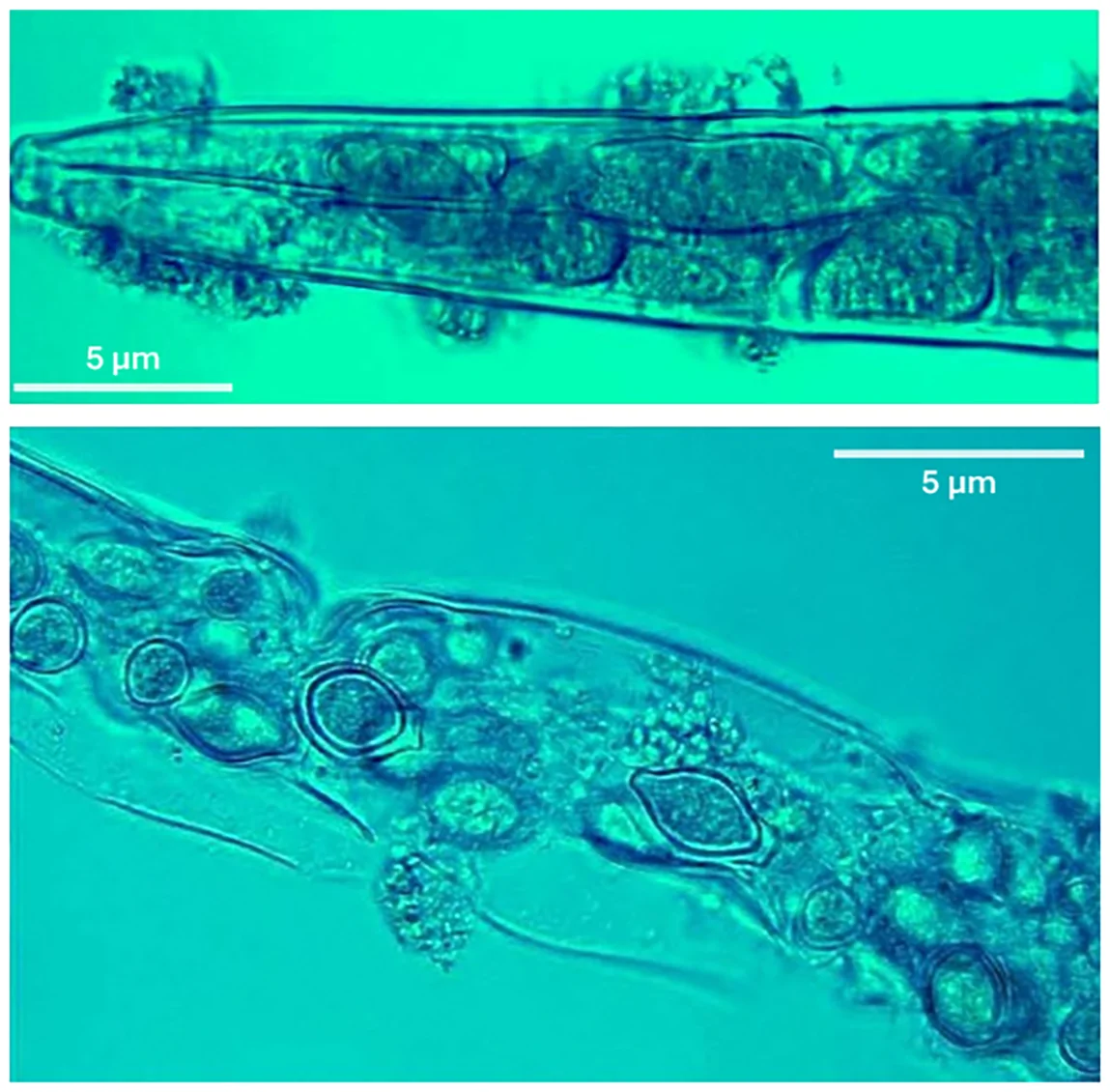
Sporangia of the aquatic fungus *Catenaria anguillulae* within specimens of *Xiphinema* sp. The sporangia of this ubiquitous aquatic fungus form inside the host. When mature, they release zoospores that swim and attach to new nematodes. The zoospores then encyst, lose their flagella, and germinate penetrating the host cuticle to originate a vegetative coenocytic mycelium that digests the nematode content, completing the cycle with the formation of new sporangia.

However, many PPNs are naturally regulated by aquatic fungi and fungus-like protists (primarily Chytridiomycota, Rozellomycota, and Oomycota, e.g. *Myzocitium* spp.). Their zoospores serve as specialized attachment units that, upon losing the flagellum, adhere to the host cuticle and differentiate into an infectious cyst. This encysted zoospore germinates by penetrating the host cuticle, developing a coenocytic mycelium (aseptate hyphae). Species such as *Catenaria anguillulae* are endemic to almost any saturated soil or aquatic environment, forming an integral part of the natural nematode-regulating microbial complex (**Figure 3**). While aquatic fungi contribute to pest suppression, their biocontrol potential is constrained by limited host specialisation and a strict dependence on high soil moisture content.

Across all NF groups, nematode parasitism culminates in the complete degradation and assimilation of the prey or egg, providing the fungus with essential nutrients and metabolic energy for vegetative growth. Host recovery following a predatory or parasitic attack is exceptionally rare, with host mortality being the inevitable outcome. Hydrolytic enzymes secreted by NFs (predominantly serine proteases, chitinases, glucanases, collagenases) play a central role in breaching the structural barriers of the cuticle and facilitating internal digestion of the nematode (Meel and Saharan, 2025).

## 2 Evolution and phylogenetic relationships

Several NFs have been reclassified under updated binomial names following relevant taxonomic revisions and nomenclatural standardisation (e.g. *Monacrosporium* renamed to *Drechslerella*, *Dactylella* to *Hyalorbilia*, *Verticillium* to *Haptocillium*) (Gams and Zare, 2001; Zare and Gams, 2001; Chen et al., 2007; Baral et al., 2018).

NFs constitute a complex, ecologically pivotal group of soil microorganisms, defined by their ability to capture and exploit nematodes as a nutrient source. This predatory lifestyle relies on the differentiation of highly specialized trapping structures, whose morphology and biomechanical mode of action are critical to fungal survival. Historically, these functional traits were considered direct indicators of evolutionary relationships. The diversity of capture devices and their underlying phylogenetic lineages were thought to provide key insights into the evolutionary trajectories that drove the emergence of predation in fungi, positioning these organisms within the broader evolutionary framework of invertebrate-pathogenic fungi.

Capture devices were considered to provide more informative phylogenetic evidence than asexual reproductive structures (conidiophores and conidia). Being highly differentiated morphological traits essential for survival, these structures remain inducible even after prolonged subculturing on artificial media (Yang et al., 2007). Trapping structures in predatory fungi play a key role in nutrient acquisition and confer significant competitive advantages within the soil microcosm over non-predatory fungi; consequently, they were regarded as adaptations shaped by strong functional selective pressures (Rubner, 1996; Scholler et al., 1999).

Most NF species capable of capturing nematodes possess a teleomorph within the genus *Orbilia*. Traditionally, their taxonomic classification was organized primarily according to trap morphology (Ahrén et al., 1998). Five distinct types of capture devices have been recognized and characterised in predatory fungi linked to *Orbilia* (Ascomycota) (Yang and Liu, 2006; Yang et al., 2007). Among these, the adhesive network (I) is initiated by an short lateral branch extending from a vegetative hypha that loops and fuses back with the parent hypha. Further interconnected loops subsequently develop from the original structure or adjacent hyphae. This architecture represents the most widespread trapping mechanism and is characteristic of the genus *Arthrobotrys*.

The adhesive knob (II) consists of a morphologically distinct globose or subglobose cell, which may be sessile on the vegetative hypha or borne on an erect peduncle. The adhesive knobs are closely spaced along the hyphae and are characteristic of several species within the genus *Dactylellina*.

Non-constricting rings (III) often occur alongside adhesive knobs; they are formed when a lateral branch extends from a vegetative hyphae, curves, and fuses with its own supporting peduncle to yield a three-celled ring structure.

The adhesive column (IV) is a short, erect branch composed of a few swollen cells arising from the hypha; this structure occurs in species such as *D. cionopaga*, *D. gephyropaga*, *D. robusta* and *D. arcuata*. All these capture devices immobilise nematodes via an adhesive matrix coating all or part of their surfaces.

Finally, the constricting ring (V), the most sophisticated mechanical trapping mechanism, intercepts prey dynamically. When a nematode enters the constricting ring aperture, the three ring-forming cells rapidly inflate inward, securely trapping the host within 1–2 seconds. This mechanism is characteristic in *Drechslerella* spp (Yang et al., 2007).

Rubner (1996) hypothesized that predatory fungi evolved from non-predatory ancestors and proposed that the least differentiated capture device was the sessile adhesive knob, from which three distinct evolutionary lineages subsequently diverged: (I) adhesive hyphal columns, which further differentiated into two- and ultimately three-dimensional scalariform networks; (II) adhesive stalked knobs, non-constricting rings and constricting rings; and (III) the adhesive columns featuring globose terminal cells with proliferating knobs. On this basis, the author suggested that constricting rings and 3D adhesive networks represent the most highly evolved trapping organs, reflecting their wide distribution (Rubner, 1996).

Conversely, Li et al. (2005) proposed an alternative model in which the adhesive network is regarded as an ancestral, less evolved device due to its lower capture efficiency. Furthermore, certain fungi, such as *A. anomala* and *A. botryospora*, form rudimentary adhesive networks analogous to the traps observed in *Cystopage* spp. and *Stylopage* spp. (Zoopagomycota) (Barron and Davidson, 1972). Under this alternative model, the adhesive network may have undergone simplification into an adhesive column, which subsequently evolved into non-constricting and constricting rings via intermediates such as the hyphal column or the pedunculated knob.

Constricting and non-constricting rings appear closely related at first glance due to their gross morphological and functional similarities. However, these systems rely on distinct capture mechanisms and differ fundamentally in their ontogenetic development. Ribosomal RNA (rRNA) sequence analysis have revealed that species forming non-constricting rings are phylogenetically distant from those with a constricting ring; instead, they are closely aligned with taxa producing pedicle adhesive knobs. The adhesive column appears to represent one of the more evolutionary derivates of primitive adhesive cells. Three-dimensional adhesive networks closely resemble vegetative hyphae coated with an extracellular sticky matrix, whereas two-dimensional networks exhibit marked constriction at the septa, structurally mirroring adhesive column (Li et al., 2005).

Compared to sessile knobs, the adhesive column and non-constricting rings provide a significantly larger surface area for nematodes attachment. Species capable of producing non-constricting rings frequently co-produce pedicle adhesive knobs. When captured nematodes struggle to break free, both the adhesive knob and the non-constricting ring can detach at their basal hyphal connection (Yang et al., 2007). The detached structures afford a distinct evolutionary and epidemiological advantage to NFs, as they can be carried passively by the swimming or migrating nematode within soil matrix. While remaining firmly bound to the cuticle, these detached propagules limit host mobility, subsequently germinate to penetrate the body, and enable fungal proliferation alongside spatial dissemination.

Phylogenetic reconstruction based on rRNA gene sequences supports the hypothesis that fungi sharing broad capture strategies (i.e., mechanical *vs* adhesive) belong to the same evolutionary lineage (Yang et al., 2007). At macro-evolutionary scale, ribosomal gene data delineate two primary evolutionary lineages corresponding to these different strategies: a non-adhesive lineage comprising exclusively constricting-ring species, and a second lineage encompassing all taxa relying on adhesive structures (e.g., adhesive branches, knobs, networks).

However, within these major lineages, and particularly among specific adhesive trap types, the evolutionary history of trapping fungi represents a paradigm shift in fungal systematics, wherein morphology-based hypotheses have been critically re-evaluated in the light of molecular phylogenetics. The polyphyly observed among superficially similar traps (such as non-constricting versus constricting rings, or various adhesive structures) underscores how strong functional selective pressures within the soil microcosm have driven independent ontogenetic pathways. Consequently, while primary capture mechanisms align with major clades, specific trap morphology alone cannot serve as an absolute taxonomic marker; rather, it represents a highly adaptive trait shaped by soil micro-ecological niches and predator–prey co-evolution. A major open challenge facing researchers is investigating the multi-trophic interactions that regulate trap morphogenesis within natural soil matrices, where interspecific competition and physical soil parameters strongly modulate fungal persistence and predatory efficiency. Future research must prioritize integrated multi-omics approaches alongside ecological validations to further unravel the evolutionary dynamics of these predatory fungi.

Generally, entomopathogenic fungi (EFs) and NFs are facultative parasites, implying low specificity and consequently a broad host range. They can colonise a wide array of habitats and are distributed worldwide (Lopez-Llorca et al., 2008). Moreover, EFs and NFs share several similarities, including spending most of their saprotrophic growth phase as soil dwellers.

Many fungi in the family Clavicipitaceae (Ascomycota, Hypocreales) are pathogenic to invertebrates as EFs and NFs, and their interactions have been extensively investigated (Lin et al., 2015). Multi-gene phylogenetic analysis resolved Clavicipitaceae *sensu lato* into three distinct lineages within Hypocreales (Sung et al., 2007b), which were subsequently recognized as separate families (Sung et al., 2007a). The Clavicipitaceae include *P. chlamydosporia* (formerly *Verticillium chlamydosporium*, teleomorph *Metacordyceps chlamydosporia*), a widespread terrestrial species found in PPN-suppressive soils worldwide. *Pochonia chlamydosporia* is also an endophyte and can parasitise females and eggs of endoparasitic plant nematodes as a facultative hyperparasite (Escudero and Lopez-Llorca, 2012; Manzanilla-López et al., 2013).

The phylogenetic placement of the genus *Pochonia* within the family Clavicipitaceae has been inferred from selected nuclear genes. Based on a phylogenetic analysis, *P. chlamydosporia* was found to be closely related to *Metarhizium* spp. (fungi known to be entomopathogenic) (Larriba et al., 2014). *Pochonia chlamydosporia* is particularly close to *Metarhizium anisopliae*. These two species form a clade with five other entomopathogenic fungi, namely *Cordyceps brongniartii*, *Cordyceps militaris*, *Beauveria pseudobassiana*, *B. bassiana*, and *Akanthomyces muscarius* (Lin et al., 2015). Recent metabolomic studies are consistent with the phylogenetic closeness between *P. chlamydosporia* and the genus *Metarhizium*, reinforcing the hypothesis that certain species of *Metarhizium* and *Pochonia* share a common ancestor (Lozano-Soria et al., 2020).

## 3 Metabolites produced by nematophagous fungi

Fungi are a rich source of bioactive secondary metabolites. However, a significant portion of fungal chemical diversity remains underexplored, particularly in nematode-associated species (Llanos-López et al., 2026). Fungal secondary metabolites play key roles in biological processes and ecological interactions, offering promising applications across several fields, including invertebrate biocontrol, where they act directly on pests and/or stimulate plant defences (Rincy et al., 2025). In this context, NFs represent a particularly promising resource not only for crop protection, but also for the discovery of bioactive molecules with potential applications in the biomedical field.

Among the mechanisms through which these microorganisms exert their antagonistic action towards nematodes, antibiosis plays a primary role. This process is mediated by the production of several secondary metabolites and extracellular enzymes, including xylanases, pectinases and glucanases, which contribute to nematotoxicity (Vos et al., 2012). Extracellular enzymes can degrade the eggshell and cuticle of nematodes, facilitating fungal penetration and access to nutrients within host tissues. Furthermore, numerous NFs produce VOCs, which can influence the behavior, survival, and distribution of PPNs and other pests. In this way, they open new research perspectives for the development of more sustainable biological control strategies and for reducing reliance on synthetic chemical pesticides (Pereira et al., 2026).

Several metabolites produced by fungal isolates of the genera *Orbilia*, *Arthrobotrys*, and *Pochonia* are active against PPNs. Among these, benzaldehyde and 1,3-dimethoxybenzene exhibited high nematicidal activity against *Pratylenchus brachyurus*, as shown by *in-vitro* and *in-vivo* assays (Pereira et al., 2026). VOCs production highlights their potential as natural nematicides, alone or in combination with other biological control agents. Metabolites produced by *Purpureocillium lilacinum* and *P. variotii* have been shown to be effective in controlling *M. incognita*, inhibiting egg hatching, and causing mortality in juveniles, with dose-dependent effects linked to the metabolite structural complexity (Patidar et al., 2024). The presence of numerous nematicidal metabolites, particularly fatty acids produced especially by *P. lilacinum*, was positively correlated with nematicidal efficacy. Such nematicidal metabolites can be exploited to develop environmentally friendly, low-cost biocontrol strategies that demonstrate comparable efficacy to synthetic chemical nematicides (Patidar et al., 2024).

Several VOCs produced by *P. chlamydosporia* and *M. robertsii* showed repellent activity against the black banana weevil, *Cosmopolites sordidus* (Coleoptera: Dryophthoridae) (Lozano-Soria et al., 2020; 2024). Specifically, 1,3-dimethoxybenzene interferes with the attraction of *C. sordidus* towards its aggregation pheromone in field trials conducted on banana crops. These data suggest the feasibility of incorporating these metabolites into integrated “push–pull” management strategies for the sustainable control of *C. sordidus* and other agricultural pests (Lozano-Soria et al., 2025).

The production of secondary metabolites in NFs can be induced *in vitro* by exposing the mycelium to various biotic and abiotic stimuli. For example, *P. chlamydosporia*, being resistant to chitosan, can degrade and use this polymer as a nutrient source. Chitosan is a biopolymer that also functions as an elicitor of plant defences pathways and can significantly modulate the secondary metabolome of *P. chlamydosporia*, inducing the synthesis of specific organic compounds and modifying their relative quantities (Mestre-Tomás et al., 2023). Compounds such as 3-methylbutanoic acid, 1-octen-3-ol, and tetradec-1-ene are produced by *P. chlamydosporia* exclusively in the presence of chitosan. Among these, 3-methylbutanoic acid is a known effective fungicide against the phytopathogenic fungus *Alternaria alternata* (Wang et al., 2022), whereas 1-octen-3-ol is recognized as a repellent against several phytophagous organisms (Wallingford et al., 2017; Lozano-Soria et al., 2020).

In addition to NF-derived metabolites, the application of EF metabolites for controlling PPNs is gaining increasing attention. For instance, beauvericin produced by *B. bassiana* and *B. pseudobassiana*, or by certain *Fusarium* spp., has demonstrated clear nematicidal activity against *Bursaphelenchus xylophilus* and *Caenorhabditis elegans* (Shimada et al., 2010; Sánchez-Gómez et al., 2023).

The chemical diversity of nematode-associated fungi is further highlighted by the isolation of seven novel natural products from *Polydomus karssenii*, characterised by antimicrobial, antifungal, and nematicidal activities, underscoring the remarkable potential of these microorganisms as a source of novel bioactive molecules that remain largely unexplored (Llanos-López et al., 2026). Similarly, *P. chlamydosporia* produces several metabolites of interest, including enniatins, pseudotin A, and viridin, which exhibit antifungal and nematicidal activities (Rincy et al., 2025). Certain metabolites produced by fungal species, such as bisevertinol and enniatins, also exhibit cytotoxic and antitumor properties, further broadening the application prospects of these secondary metabolites in biomedical research (Rincy et al., 2025).

## 4 Nematophagous fungi applications

NFs are an integral component of soil and rhizosphere microbial communities, where they participate in the assembly of complex food webs. Within such ecological systems, numerous species interact through functional relationships of varying nature, which can be cooperative (as mutualistic symbiosis or endophytism) or antagonistic (such as predation, parasitism, and competition). Soil invertebrates are also embedded within these networks, with PPNs representing a significant component. The interplay of these interactions contributes to the delivery of key ecosystem services, including nutrient recycling, nitrogen fixation, organic matter decomposition, and the natural regulation of plant pest populations. These processes directly or indirectly support plant growth and productivity (Mercado-Blanco et al., 2018).

Although NFs are widely distributed across soils in diverse ecosystems, many species do not exert a direct role in controlling PPNs, as they predominantly prey upon free-living nematodes or affect only a limited fraction of the parasite populations. Despite this limitation, they perform a crucial ecological role in soil food webs, contributing to the regulation of microbial communities and the decomposition of organic matter, thereby facilitating nutrients recycling.

Many biocontrol agents are investigated solely under the laboratory conditions and exhibit variable efficacy in the field due to fluctuating environmental parameters. Major bottlenecks include mass production, formulation, shelf-life preservation, application technology, and farmers awareness, all of which necessitate specialized training and technological advancements (Shukuru et al., 2024). Currently, PPN management relies on an integrated framework of strategies encompassing regulatory quarantine measures, agronomic practices (such as crop rotations and the use of nematode-free propagating material), physical interventions (such as plowing, solarisation, or heat treatments), and chemical nematicides (Meel and Saharan, 2025). However, biological control applications using fungi remain largely limited to a few NF species that suppress PPN populations through direct parasitism or the secretion of toxic metabolites. In this context, NFs represent promising tools for developing sustainable plant protection strategies capable of reducing reliance on synthetic chemical nematicides and promoting soil health.

Among the microorganisms most extensively studied for applied purposes are *P. chlamydosporia* and a few other hyphomycetes. Various isolates of *P. chlamydosporia* have demonstrated high persistence and efficacy in the biological control of nematodes under both greenhouse and field conditions. Furthermore, *P. chlamydosporia* is multitrophic and can colonise plant roots as an endophyte in both monocotyledonous and dicotyledonous species (Manzanilla-López et al., 2013). Endophytic colonisation by *P. chlamydosporia* interacts positively with plants performance, enhancing vegetative growth and resistance by activating specific pathways involved in systemic plant defence response (Pentimone et al., 2019; Tolba et al., 2021; Curci et al., 2025; Shaliha et al., 2025). The multitrophic transition in *P. chlamydosporia* is linked to the expression of enzymes that degrade polysaccharides in both the plant cell wall and nematode eggshell, including glycosidases and serine proteases (Berosich et al., 2024).

Applications of *P. chlamydosporia* have mainly focused on nematode biocontrol. A long-term experiment conducted at Rothamsted Research in the United Kingdom demonstrated that *P. chlamydosporia* acts as a natural regulator of the cereal cyst-nematode, *Heterodera avenae*. Soil treatment with formalin, which is lethal to the fungus but not to the nematode, resulted in a significant increase in nematode population density, highlighting the antagonist’s regulatory role. This work represents one of the earliest examples of how long-term field studies can clarify the ecological role of microbial antagonists within the rhizosphere and their effects on pathogen population dynamics and crop productivity (Kerry et al., 1982; Kerry and Crump, 1998).

Subsequent studies have highlighted a close association between specific *P. chlamydosporia* isolates and the nematode species with which they coexist in nature, suggesting co-evolutionary phenomena. This adaptation involves the VCP1 enzyme, a serine protease key to degrading the nematode cuticle. Polymorphisms in the VCP1 coding sequence, arising from point mutations, alter its amino acid sequence and consequently three-dimensional structure of the encoded protein, thereby conferring greater specificity for the cuticle of the preferred host. Analysing of VCP1 gene polymorphisms therefore enables the identification of optimal targets and the selection of the most suitable isolates for controlling specific nematodes groups, such as root-knot or cyst nematodes (Morton et al., 2003). *Pochonia chlamydosporia* exhibits a tritrophic behavior comprising nematode egg parasitism, root endophytism, and saprotrophic metabolism. The integration of these different lifestyles contributes both to the biological control of nematodes and to the promotion of plant growth (Manzanilla-López et al., 2013). However, the mechanisms regulating transition between these trophic modes are not yet fully understood. Results of tripartite experiments investigating the interaction between *P. chlamydosporia* and *Meloidogyne javanica* in presence of chitosan highlighted considerable phenotypic plasticity in *P. chlamydosporia* during the saprotrophic-to-parasitic transition. Chitosan acts as a metabolic and fungal virulence stimulator, promoting a proteomic profile characteristic of “parasitic readiness” and increasing the abundance of carbohydrate-active enzymes (CAZymes) and extracellular hydrolases that facilitate the parasitism of nematode eggs. The fungus employs a “host detection program” in which chemical signals from nematode eggs trigger and modulate the germination of resting propagules. Concurrently, chitosan destabilises the nematode resistance pathways related to reproduction (via perturbation of vitellogenin), structural integrity (via suppression of essential eggshell components) and metabolic stress (Berosich et al., 2026).

Other factors influencing the tripartite saprotrophic/parasitic/endophytic transition of *P. chlamydosporia* include nutrient availability and root exudates, which are known to modulate fungal growth and behavior. For instance, high concentrations of carbon-rich compounds can inhibit the production of enzymes involved in infectious processes, whereas high soil organic matter levels promote fungal growth without necessarily enhancing biocontrol efficacy (Kerry and Bourne, 1996). Gene expression analysis have further demonstrated that unfavorable environmental conditions, such as easily metabolised carbon sources or sub-optimal pH values in the rhizosphere or nematodes egg masses, can compromise parasitism by *P. chlamydosporia* (Ward et al., 2012; Luambano et al., 2015). Transcriptomic studies have revealed marked gene expression reprogramming in *P. chlamydosporia*, in response to varying nutritional conditions, indicating the activation of specific gene clusters during transitions between trophic modes (Rosso et al., 2011). Among the expressed genes, a phytase gene was identified whose transcription increases dramatically in the presence of root-knot nematode eggs (with levels up to 900 times higher than in the control), suggesting a potential role of the fungus also in phosphorus metabolism (Rosso et al., 2014).

The metabolism of *P. chlamydosporia* therefore plays a central role in its rhizosphere interactions. Several proteins secreted by the fungus are involved in responding to nutrient deficiency, primarily proteases and glycosidases (Lin et al., 2018). Studies on interactions with *Arabidopsis* have highlighted jasmonates as main regulators of endophytic colonisation (Zavala-Gonzalez et al., 2017). During the colonisation of barley roots, the fungus produces numerous proteases, hydrolases and transport proteins (Lin et al., 2018). Furthermore, endophytic colonisation of tomato roots by *P. chlamydosporia* influences the expression of specific host-plant microRNAs and their target genes, which are involved in signal perception and transduction (Pentimone et al., 2018).

The tritrophic activity of *P. chlamydosporia* does not necessarily represent a constraint on its application. Model-based simulations of the interactions between *P. chlamydosporia* and *M. incognita* have revealed an indirect mechanism for regulating nematode populations, linked to an increased fungal biomass resulting from saprotrophic growth. Modelling showed that the fraction of saprotrophic mycelium present in the soil can contribute to biological control following transition to a parasitic lifestyle, provided it exceeds a daily biomass growth rate threshold of 0.02% (Ciancio et al., 2022). Therefore, the portion of mycelium not directly involved in parasitism represents an ecological reserve capable of sustaining subsequent cycles of infection or endophytic colonisation.

Endophytes play a fundamental role in plant physiology and can significantly influence host growth and productivity. In many cases, they promote plant development by activating systemic defensive responses against biotic and abiotic stresses. In *P. chlamydosporia*, root colonisation induces root metabolic shift, including an accumulation of phenolic compounds and flavonoids, enhanced uptake of nutrients such as phosphorus. Currently, it triggers the activation of host defence-related genes, generating an early protective response effective against root pathogens, including root-knot nematodes (Pentimone et al., 2019; Shaliha et al., 2025). In tomato roots, the presence of *P. chlamydosporia* led to changes in the expression of gene associated with stress responses and the jasmonic acid pathway, including the activation of lipoxygenase genes (Pentimone et al., 2019). The jasmonic acid pathway is closely linked to mechanisms of induced systemic resistance against nematode invasion (Nahar et al., 2011; Ozalvo et al., 2014). Furthermore, inoculation of *P. chlamydosporia* significantly improves drought tolerance in soybeans, delaying the decline in leaf water potential and increasing water-use efficiency. Modulation of osmolytes, increased root hydraulic conductivity, alongside metabolic and anatomical roots adaptations, contribute to mitigating water-deficit stress (Mendonça Rodrigues et al., 2024).

Recently, a seed treatment formulation featuring *P. chlamydosporia* blastospores was developed, employing biopolymers such as xanthan gum and gellan gum, in combination with potato starch and bentonite to coat *Phacelia tanacetifolia* seeds. Applying biocontrol agents via seed treatment enables targeted delivery directly to the developing rhizosphere, saving time and costs relative to alternative formulation strategies. This *P. tanacetifolia* seed coating maintained blastospores viability during refrigerated storage for at least 42 days (Uthoff et al., 2024a). Biological enhancement of *P. tanacetifolia* through seed coating with *P. chlamydosporia* blastospores effectively suppressed *Meloidogyne hapla* infection (Uthoff et al., 2024b).

In addition to *P. chlamydosporia*, numerous other endophytic fungi with nematophagous activity have been reported in several wild and cultivated plants, including species belonging to the genera *Metarhizium*, *Trichoderma*, *Clonostachys*, and *Fusarium*. The evolutionary mechanisms driving the development of this dual lifestyle (endophytism and invertebrate parasitism) are not yet fully understood. It has been shown that endophytic *M. robertsii* can transfer nitrogen derived from parasitised insect to the host plant, effectively returning a portion of the nutrient removed by pests (Behie et al., 2012). Such direct nutrient transfer suggests the existence of complex mutualistic relationships between host plants and fungal endophytes.

The genus *Trichoderma* includes several species with high potential for controlling soil-borne pathogens and PPNs. *Trichoderma* spp. can act through mycoparasitism, antibiosis and competition, while concurrently promoting plant growth (Spiegel and Chet, 1998; Sharon et al., 2001, 2007; Kumar and Jain, 2010; Olmedo-Monfil and Casas-Flores, 2014; López-Bucio et al., 2015; Alhazmi et al., 2025). For example, *T. harzianum* has been successfully deployed against PPNs such as *M. javanica* (Sharon et al., 2001, 2007) and *Globodera pallida* (Dandurand and Knudsen, 2016; Contina et al., 2017). Similarly, *T. longibrachiatum* exhibited direct *in vitro* activity against eggs and juveniles of *H. avenae* and *M. incognita*, an efficacy subsequently validated under greenhouse conditions through significant reduction in nematode populations (Zhang et al., 2017; Alhazmi et al., 2025).

Finally, another endophyte group with promising application prospects is represented by the genus *Clonostachys*. *Clonostachys rosea* is a well-known mycoparasite and antagonist of several soil-borne pathogens, including root-knot nematodes (Verdejo-Lucas et al., 2002; Sun et al., 2020; Stucky et al., 2024). Furthermore, *C. rogersoniana* can penetrate and degrade *Meloidogyne paranaensis* eggs *in vitro* (Lima-Rivera et al., 2025), highlighting the potential of these endophytic fungi for sustainable crop protection strategies.

The pioneering application of NFs in nematode control has recently expanded to include their use as an efficient and sustainable vehicles for delivering functional RNA molecules. RNA interference (RNAi) represents a highly targeted and promising strategy for PPNs management. Effective delivery of RNA molecules into nematodes was achieved via extracellular vesicles (EVs) derived from engineered *A. oligospora* strains. These engineered EVs efficiently transported RNA cargoes into nematodes, leading to significant downregulation of crucial nematode neuropeptide genes (flp and nlp gene families) in *M. incognita*, alongside marked reductions in root invasion and infectivity. This study provided the first demonstration that EVs from a NF can serve as effective RNA delivery vehicles for root-knot nematode control (Huang et al., 2025).

Furthermore, *A. oligospora* was recently employed in the green synthesis of zinc oxide nanoparticles (ZnO-NPs) using its extracellular filtrate under controlled conditions. The biogenic ZnO-NPs synthesised via *A. oligospora* exhibited nematicidal activity against *M. incognita*, offering an eco-friendly and multifunctional alternative to synthetic chemical nematicides (Gautam et al., 2025).

## 5 Isolation, production and bioformulates

Isolation of NFs from soil can be performed using relatively simple techniques based on their ability to develop in the presence of nematodes. In the case of trapping fungi, it is often sufficient to disperse small soil fragments of medium onto water agar (1.5%) and await the proliferation of microbivorous nematodes presents within the inoculum. Nematodes population growth subsequently stimulates the development of their fungal antagonists, a process that generally occurs over one to two weeks. Trapping fungi can be identified by their characteristic hyphal trapping structures that form on the agar surface. Monoconidial isolates can then be established by collecting conidia from the apex of conidiophores emerging from the substrate, streaking them onto nutrient media, and subsequently subculturing the smallest colonies that develop over the following days.

Endoparasitic fungi, on the other hand, can be isolated from infected nematode corpses or parasitised eggs. Nematodes are typically recovered from suspensions obtained via soil filtration and are often quantified using volumetric counting chambers, such as the Hawksley chamber. Individual specimens can be hand-picked under a stereomicroscope using a fine needle equipped at its tip with a bristle, an eyelash, or a thin filament, and subsequently transferred onto water agar. Following a few days of incubation at room temperature, the emergence of fungal hyphae can be observed, from which the actively growing hyphal tips or conidia produced on conidiophores can be sampled. Similar procedures allow the isolation of *P. chlamydosporia* or other hyphomycetes by harvesting conidia borne along aerial hyphae that develop on the agar. These structures generally emerge within a few days from soil particles, individual eggs, or nematode egg masses, as well as from surface-sterilized root fragments in the case of endophytic fungi. Alternatively, eggs already infected with endoparasites can be dispersed onto water agar to wait hyphal emergence.

Specific micro-tools are employed to harvest conidia, consisting of fine tungsten filaments salvaged from incandescent light bulbs or eyelashes/bristles attached to needle tips. These instruments are sterilised by brief immersion in ethanol and left to air-dry away from air currents or, in the case of tungsten filaments, flame-sterilised directly. The collected conidia should be transferred onto a nutrient substrate, such as Potato Dextrose Agar (PDA), by touching the tip of the micro-tool to the agar surface and tracing an S-shaped streak. Over the following days, colony development can be monitored along the streak line; the smaller, well-isolated colonies can generally be considered to originate from a single conidium and can be subcultured by transferring a marginal hyphal fragment to obtain a definitive monoconidial isolate.

In recent years, several commercial products based on NFs have been developed for the biological control of PPNs and for promoting plant growth in intensive and peri-urban agricultural systems. The simplest formulations consist of aqueous suspensions containing fungal inocula, such as *P. chlamydosporia*, which can be applied directly to the soil or root system, maintaining soil moisture close to field capacity to favor the establishment of the microorganism (Ciancio et al., 2016).

In Italy, a commercial formulation containing microspores of *P. chlamydosporia* and *A. oligospora* (both at 10^8^ CFU/g) was developed and marketed for application via fertigation. The product was designed to amend soil by introducing a consortium of beneficial microorganisms to stabilise the population dynamics of *Meloidogyne* spp. The aim is to promote the restoration of soil ecological balance, particularly under conditions where the native microflora has been altered by previous chemical fumigations or other intensive management practices. The formulation also included inocula of arbuscular mycorrhizal fungi of the genus *Glomus* and plant growth-promoting rhizobacteria of the genus *Bacillus*. This microbial consortium contributes to the management of root-knot nematodes as well as the nutrition and health of the plant root system (Sellitto et al., 2016).

A further approach involves the application of *P. chlamydosporia* in combination with winemaking by-products. Field studies have demonstrated that this combined strategy suppresses *M. javanica* populations while enhancing plant growth. Grape residues are rich in nematicidal compounds, including polyphenols, organic acids (such as gallic, caffeic, ferulic and p-coumaric acids), and the stilbene trans-resveratrol (Oka et al., 2007; Reiner Driéli et al., 2016). These substances can inhibit egg hatching or cause mortality in second-stage juveniles (J2). Furthermore, eggs partially degraded by these compounds appear to be more susceptible to parasitism by *P. chlamydosporia*, making this approach a promising eco-friendly alternative to chemical nematicides in greenhouse cropping systems (Dalla Pasqua et al., 2020).

Innovative formulations based on oil dispersions containing mycelial fragments and conidia and of *P. chlamydosporia* emulsified with vegetable oils have also been developed. A formulation with a concentration of 3.3 × 10^8^ CFU/ml reduced root-knot nematode infestations, exhibiting storage stability for up to 90 days at room temperature (Swarnakumari et al., 2020).

Commercial products already available include the bionematicide KlamiC^®^, based on *P. chlamydosporia* var. *catenulata* (selected strain IMI SD 187). This formulation has demonstrated high efficacy in controlling *Meloidogyne* spp. Following the application, *P. chlamydosporia* rapidly colonises the soil and root systems, parasitising target nematodes. In addition to its nematicidal activity, *P. chlamydosporia* var. *catenulata* has been reported to promote root development and enhance fertiliser absorption, while stimulating phytohormone production and sustaining induced systemic resistance in the host plant. The product is obtained via solid-state fermentation in polypropylene bags equipped with gas-exchange filters (Hernández Socorro et al., 2016).

The efficacy of certain biocontrol agents, including *P. chlamydosporia* and *B. bassiana*, can be further enhanced by combining them with chitosan. As a biological agricultural additive, chitosan possesses antifungal and antiviral activities, promotes plant tolerance to abiotic and biotic stresses, and stimulates conidiogenesis in NFs (Lopez-Nuñez et al., 2025). In soils where *P. chlamydosporia* is naturally present or inoculated, chitosan application promotes greater root colonisation and enhance nematode eggs parasitism. This approach represents a sustainable strategy for suppressing PPN populations in agroecosystems, effectively aligning with circular economy and sustainable agriculture paradigms (Lopez-Nuñez et al., 2022). Furthermore, direct soil application of chitosan stimulates sporulation and competition from NFs such as *Purpureocillium* spp., thereby potentially increasing their biocontrol efficacy while suppressing the abundance of susceptible fungal phytopathogens, such as *Fusarium* spp. and *Alternaria* spp (Lopez-Nuñez et al., 2025).

Other soil fungi have also been developed as commercial bionematicides. In the United States, for example, products based on *P. lilacinum* (formerly *Paecilomyces lilacinus*) and *Myrothecium verrucaria* are commercially available. These formulations contain fungal spores or fermentation products, respectively, and can parasitise or inhibit a wide range of PPNs, including root-knot, root-lesion, and cyst nematodes (Arthurs and Dara, 2019). These products are registered for numerous crops and can serve as partial alternatives to synthetic chemical soil fumigants (Dong et al., 2015; Baidoo et al., 2017).

Finally, many *Trichoderma* species are widely used in commercial bioformulations designed for plant protection and growth promotion (Harman et al., 2004; Lorito et al., 2010; Oskiera et al., 2015). Several isolates of *Trichoderma* spp. produce metabolites and enzymes with antimicrobial and biocontrol activities, while also colonising roots to induce host metabolic changes that enhance resistance to phytopathogens attacks (Vinale et al., 2006, 2012). Among the metabolites produced by *Trichoderma* spp. are mycotoxins belonging to the trichothecene group, compounds that have attracted considerable attention due to their biological effects on plants, animals, and humans (McCormick et al., 2011; Malmierca et al., 2012; 2015). The production of trichothecenes and other bioactive metabolites by *Trichoderma* spp. has been linked to their antimicrobial properties and efficacy as biocontrol agents (Vizcaíno et al., 2005; Tijerino et al., 2011; Malmierca et al., 2012). The mortality of nematode juveniles observed across several experimental assays is likely associated with the production of one or more of these compounds, which are known to exert direct nematicidal effects (Ciancio, 1995; Nitao et al., 2001; Benttoumi et al., 2020).

## 6 Conclusions

Nematophagous fungi represent an extremely diverse and functionally complex group of soil microorganisms with significant application potential in the biocontrol of plant-parasitic nematodes. Their diverse attack strategies, encompassing physical, enzymatic, and chemical mechanisms, reflect evolutionary adaptations closely tailored to environmental conditions and resource availability. However, the application of these fungi is not always straightforward, as field failures can often hinder their full exploitation. The selection of best performing isolates *vs* specific nematode pests is a strategy often required to replicate laboratory results in field conditions. Several formulation challenges must be afforded for this purpose, including issues related to the isolates stability and conservation, their adaptation to different environmental niches and their competition with indigenous microbial communities. Shelf-life improvement of formulations is a further issue requiring investments in research and testing. These aspects represent indeed research gaps that need to be overcome in view of producing effective, biocontrol formulations. Despite substantial advances, the field efficacy of these organisms remains in effect variable and dependent on multiple biotic and abiotic factors, necessitating a broad availability of isolates for selection and a deeper understanding of their rhizosphere interactions, including the identification of key metabolic and genetic traits. Phylogenetic data suggest that predatory capacity evolved through convergent yet functionally distinct strategies. Looking ahead, the integration of NFs into sustainable management systems will require a multidisciplinary approach aimed at optimising their use under real agronomic conditions and developing effective, stable formulations compatible with other protection strategies.

## References

[B1] Ahrén D. Ursing B. M. Tunlid A. (1998). Phylogeny of nematode-trapping fungi based on 18S rDNA sequences. FEMS Microbiol. Lett. 158, 179–184. doi: 10.1111/j.1574-6968.1998.tb12817.x 9465391

[B2] Alhazmi N. Ibrahim D. S. S. Moussa T. A. A. (2025). Evaluation of nematicidal and plant growth promotion properties of some soil nematophagous fungi against root-knot nematode Meloidogyne incognita: *in vitro* and greenhouse studies. Egypt. J. Bot. 65, 589–600. doi: 10.21608/ejbo.2025.368405.3226

[B3] Arthurs S. Dara S. K. (2019). Microbial biopesticides for invertebrate pests and their markets in the United States. J. Invertebr. Pathol. 165, 13–21. doi: 10.1016/j.jip.2018.01.008 29402394

[B4] Arunachalam A. Ganesan S. Pandiyan V. Palaniyappan K. Nagachandrabose S. Kandasamy D. (2025). Unfastening sublethal effects of the nematophagus fungus Pochonia chlamydosporia [TNAU Pc-001] on the biotic potentials of Meloidogyne enterolobii (Yang & Eisenback 1983) (Tylenchida: meloidogynidae). J. Crop Health 77, 87. doi: 10.1007/s10343-025-01149-130311153

[B5] Baidoo R. Mengistu T. McSorley R. Stamp R. H. Brito J. Crow W. T. (2017). Management of root-knot nematode (Meloidogyne incognita) on Pittosporum tobira under greenhouse, field, and on-farm conditions in Florida. J. Nematol. 49, 133–139. doi: 10.21307/jofnem-2017-057 28706312 PMC5507133

[B6] Baral H. O. Weber E. Gams W. Hagedorn G. Liu B. Liu X. Z. . (2018). Generic names in the Orbiliaceae (Orbiliomycetes) and recommendations on which names should be protected or suppressed. Mycol. Prog. 17, 5–31. doi: 10.1007/s11557-017-1300-630311153

[B7] Barron G. L. (1977). The Nematode-Destroying Fungi. 1st Ed (Guelph, ON, Canada: Canadian Biological Publications), 1.

[B8] Barron G. L. Davidson J. G. N. (1972). Nematophagous Hyphomycetes: Arthrobotrys anomala sp. nov. Can. J. Bot. 50, 1773–1774. doi: 10.1139/b72-22034819996

[B9] Becker J. S. Borneman J. Becker J. O. (2013). Dactylella oviparasitica parasitism of the sugar beet cyst nematode observed in trixenic culture plates. Biol. Control 64, 51–56. doi: 10.1016/j.biocontrol.2012.10.00742574925

[B10] Behie S. W. Zelisko P. M. Bidochka M. J. (2012). Endophytic insect-parasitic fungi translocate nitrogen directly from insects to plants. Science 336, 1576–1577. doi: 10.1126/science.1222289 22723421

[B11] Benttoumi N. Colagiero M. Sellami S. Boureghda H. Keddad A. Ciancio A. (2020). Diversity of nematode microbial antagonists from Algeria shows occurrence of nematotoxic Trichoderma spp. Plants 9, 941. doi: 10.3390/plants9080941 32722274 PMC7465808

[B12] Berosich C. M. Lopez-Moya F. Lopez-Llorca L. V. (2024). Modulation of the host defence system by nematophagous fungi and chitosan. Encyclopedia 4, 379–394. doi: 10.3390/encyclopedia401002630654563

[B13] Berosich C. M. Valverde-Urrea M. Zubcoff J. J. Lopez-Llorca L. V. Lopez-Moya F. (2026). Machine-learning-guided proteomic profiling reveals chitosan-modulated parasitism in the Pochonia chlamydosporia–root-knot nematode interaction. Physiol. Mol. Plant Pathol. 145, 103287. doi: 10.1016/j.pmpp.2026.10328742574925

[B14] Chen J. Xu L. L. Liu B. Liu X. Z. (2007). Taxonomy of Dactylella complex and Vermispora. II. The genus Dactylella. Fungal Divers. 26, 85–126.

[B15] Chun J. Ko Y. H. Kim D. H. (2020). Transcriptome analysis of Cryphonectria parasitica infected with Cryphonectria hypovirus 1 (CHV1) reveals distinct genes related to fungal metabolites, virulence, antiviral RNA-silencing, and their regulation. Front. Microbiol. 11, 1711. doi: 10.3389/fmicb.2020.01711 32765480 PMC7379330

[B16] Ciancio A. (1995). Observations on the nematicidal properties of some mycotoxins. Fundam. Appl. Nematol. 18, 451–454.

[B17] Ciancio A. Cabrera I. M. Hidalgo-Díaz L. Puertas A. Duvergel Y. C. (2022). Modeling root-knot nematode regulation by the biocontrol fungus Pochonia chlamydosporia. Front. Fungal Biol. 3, 900974. doi: 10.3389/ffunb.2022.900974 37746233 PMC10512345

[B18] Ciancio A. Colagiero M. Pentimone I. Rosso L. C. (2016). “ Formulation of Pochonia chlamydosporia for plant and nematode management,” in Bioformulations: For Sustainable Agriculture. Eds. Arora N. K. Mehnaz S. Balestrini R. ( Springer, Berlin, Germany), 177–197.

[B19] Contina J. B. Dandurand L. M. Knudsen G. R. (2017). Use of GFP-tagged Trichoderma harzianum as a tool to study the biological control of the potato cyst nematode Globodera pallida. Appl. Soil Ecol. 115, 31–37. doi: 10.1016/j.apsoil.2017.03.01042574925

[B20] Curci P. L. Lee J. M. Tan Q. W. Mutwil M. Ciancio A. Pentimone I. . (2025). Transcriptional re-programming of defense responses in Capsicum annuum roots induced by the interaction of the endophyte fungus Pochonia chlamydosporia and the plant-parasitic nematode Meloidogyne incognita. Curr. Plant Biol. 44, 100553. doi: 10.1016/j.cpb.2025.10055342574925

[B21] Dalla Pasqua S. Dallemole-Giaretta R. dos Santos I. Reiner D. A. Lopes E. A. (2020). Combined application of Pochonia chlamydosporia and solid by-product of the wine industry for the control of Meloidogyne javanica. Appl. Soil Ecol. 147, 103397. doi: 10.1016/j.apsoil.2019.10339742574925

[B22] Dandurand L. M. Knudsen G. R. (2016). Effect of the trap crop Solanum sisymbriifolium and two biocontrol fungi on reproduction of the potato cyst nematode, Globodera pallida. Ann. Appl. Biol. 169, 180–189. doi: 10.1111/aab.1229540046247

[B23] de Freitas Soares F. E. Sufiate B. L. de Queiroz J. H. (2018). Nematophagous fungi: Far beyond the endoparasite, predator and ovicidal groups. Agric. Nat. Resour. 52, 1–8. doi: 10.1016/j.anres.2018.05.01042574925

[B24] Degenkolb T. Vilcinskas A. (2016). Metabolites from nematophagous fungi and nematicidal natural products from fungi as an alternative for biological control. Part I: metabolites from nematophagous ascomycetes. Appl. Microbiol. Biotechnol. 100, 3799–3812. doi: 10.1007/s00253-015-7233-6 26715220 PMC4824826

[B25] Devi G. (2018). Utilization of nematode destroying fungi for management of plant-parasitic nematodes - a review. Biosci. Biotechnol. Res. Asia 15, 377–396. doi: 10.13005/bbra/2642

[B26] Dong H. Zhou X. G. Wang J. Xu Y. Lu P. (2015). Myrothecium verrucaria strain X-16, a novel parasitic fungus to Meloidogyne hapla. Biol. Control 83, 7–12. doi: 10.1016/j.biocontrol.2014.12.01642574925

[B27] Escudero N. Lopez-Llorca L. V. (2012). Effects on plant growth and root-knot nematode infection of an endophytic GFP transformant of the nematophagous fungus Pochonia chlamydosporia. Symbiosis 57, 33–42. doi: 10.1007/s13199-012-0173-330311153

[B28] Gams W. Zare R. (2001). A revision of Verticillium sect. Prostrata. III. Generic classification. Nova Hedwig. 72, 329–337. doi: 10.1127/nova.hedwigia/72/2001/329

[B29] Gao X. Yin B. Borneman J. Becker J. O. (2008). Assessment of parasitic activity of Fusarium strains obtained from a Heterodera schachtii-suppressive soil. J. Nematol. 40, 1–6. PMC2586523 19259511

[B30] Garganese F. Sanzani S. M. Ligorio A. di Gennaro D. Tarricone L. Ippolito A. (2016). Effect of irrigation management on field and postharvest quality of organic table grapes. Acta Hortic. 1144, 273–278. doi: 10.17660/actahortic.2016.1144.40

[B31] Gaspard J. T. Mankau R. (1987). Density-dependence and host-specificity of the nematode-trapping fungus Monacrosporium ellipsosporum. Rev. Nématol. 10, 241–246.

[B32] Gautam V. Garg V. Meena N. Dashrath H. A. Singh K. Kumar A. . (2025). Fungal-derived ZnO nanoparticles functionalized with riboflavin and UDP-GlcNAc exhibit potent nematicidal activity against M. incognita. Sci. Rep. 15, 38427. doi: 10.1038/s41598-025-22170-4 41184368 PMC12583721

[B33] Giuma A. Y. Cooke R. C. (1972). Some endozoic fungi parasitic on soil nematodes. Trans. Br. Mycol. Soc 59, 213–218. doi: 10.1016/S0007-1536(72)80004-4

[B34] Gray N. F. Bailey F. (1985). Ecology of nematophagous fungi: vertical distribution in a deciduous woodland. Plant Soil 86, 217–223. doi: 10.1007/BF0218289630311153

[B35] Gray N. F. Lewis Smith R. I. (1984). The distribution of nematophagous fungi in the maritime Antarctic. Mycopathologia 85, 81–92. doi: 10.1007/BF0043670730311153

[B36] Gutiérrez-Medina E. Mendoza-de Gives P. Pérez-Anzúrez G. Colinas-Picazo A. Bautista-García G. A. Alonzo-Díaz M. A. . (2024). Arthrobotrys mendozadegivensis sp. nov. (Fungi: Orbiliales) from Mexico: Predatory activity and nematocidal activity of its liquid culture filtrates against Haemonchus contortus (Nematoda: Trichostrongylidae). J. Fungi 10, 888. doi: 10.3390/jof10120888 39728384 PMC11678410

[B37] Hakariya M. Masuyama N. Saikawa M. (2002). Shooting of sporidium by “gun” cells in Haptoglossa heterospora and H. zoospora and secondary zoospore formation in H. zoospora. Mycoscience 43, 119–125. doi: 10.1007/S10267020001830311153

[B38] Harman G. E. Howell C. R. Viterbo A. Chet I. Lorito M. (2004). Trichoderma species-opportunistic, avirulent plant symbionts. Nat. Rev. Microbiol. 2, 43–56. doi: 10.1038/nrmicro797 15035008

[B39] He Q. Wang D. Li B. Maqsood A. Wu H. (2020). Nematicidal evaluation and active compounds isolation of Aspergillus japonicus ZW1 against root-knot nematodes Meloidogyne incognita. Agronomy 10, 1222. doi: 10.3390/agronomy1009122230654563

[B40] Hernández Socorro M. A. Arévalo Ortega J. A. Marrero Roque D. Hidalgo Díaz L. (2016). Effect of Klamic® on growth stimulation of plantains and bananas vitroplants. Cult. Trop. 37, 168–172. doi: 10.13140/RG.2.2.25696.69120

[B41] Huang X. Zhou W. Li X. Zhang K. Q. Li J. (2025). Biocontrol of root-knot nematodes via siRNA-loaded extracellular vesicles from a nematophagous fungus Arthrobotrys oligospora. Microb. Biotechnol. 18, e70274. doi: 10.1111/1751-7915.70274 41287984 PMC12645230

[B42] Jaffee B. A. Muldoon A. E. (1995). Susceptibility of root-knot and cyst nematodes to the nematode-trapping fungi Monacrosporium ellipsosporum and M. cionopagum. Soil Biol. Biochem. 27, 1083–1090. doi: 10.1016/0038-0717(94)00240-2

[B43] Jaffee B. A. Muldoon A. E. Westerdahl B. B. (1996). Failure of a mycelial formulation of the nematophagous fungus Hirsutella rhossiliensis to suppress the nematode Heterodera schachtii. Biol. Control 6, 340–346. doi: 10.1006/bcon.1996.004321632986

[B44] Jansson H. B. Lopez-Llorca L. V. (2001). “ Biology of nematophagous fungi,” in Trichomycetes and Other Fungal Groups: Professor Robert W. (Lichtwardt Commemoration Vol.). Eds. Misrha J. D. Horn B. W. ( Science Publisher Inc, Enfield, UK), 145–173.

[B45] Kerry B. R. (2000). Rhizosphere interactions and the exploitation of microbial agents for the biological control of plant - parasitic nematodes. Annu. Rev. Phytopathol. 38, 423–441. doi: 10.1146/annurev.phyto.38.1.423 11701849

[B46] Kerry B. R. Bourne J. M. (1996). The importance of rhizosphere interactions in the biological control of plant parasitic nematodes — a case study using Verticillium chlamydosporium. Pestic. Sci. 47, 69–75. doi: 10.1002/(SICI)1096-9063(199605)47:1<69::AID-PS386>3.0.CO;2-6

[B47] Kerry B. R. Crump D. H. (1998). The dynamics of the decline of the cereal cyst nematode, Heterodera avenae, in four soils under intensive cereal production. Fundam. Appl. Nematol. 21, 617–625.

[B48] Kerry B. R. Crump D. H. Mullen L. A. (1982). Studies of the cereal cyst-nematode, Heterodera avenae under continuous cereals 1975–1978. II. Fungal parasitism of nematode females and eggs. Ann. Appl. Biol. 100, 489–499. doi: 10.1111/j.1744-7348.1982.tb01415.x40046247

[B49] Khan A. Haris M. Khan A. A. (2023). Bio-organics management: novel strategies to manage root-knot nematode, Meloidogyne incognita pest of vegetable crops. Gesunde Pflanzen 75, 193–209. doi: 10.1007/s10343-022-00679-230311153

[B50] Krif G. Lahlali R. El Aissami A. Laasli S. E. Mimouni A. Dababat A. A. . (2024). Potential effects of nematophagous fungi against Meloidogyne javanica infection of tomato plants under *in vitro* and *in vivo* conditions. J. Crop Health 76, 829–839. doi: 10.1007/s10343-024-00989-730311153

[B51] Krijgsheld P. Bleichrodt R. van Veluw G. J. Wang F. Müller W. H. Dijksterhuis J. . (2013). Development in *aspergillus*. Stud. Mycol. 74, 1–29. doi: 10.3114/sim0006 23450714 PMC3563288

[B52] Krstin L. Katanić Z. Repar J. Ježić M. Kobaš A. Ćurković-Perica M. (2020). Genetic diversity of Cryphonectria hypovirus 1, a biocontrol agent of chestnut blight, in Croatia and Slovenia. Microb. Ecol. 79, 148–163. doi: 10.1007/s00248-019-01377-9 31053974

[B53] Kumar V. Jain R. K. (2010). Management of root-knot nematode, *Meloidogyne incognita* by *Trichoderma viride*, *T. harzianum* and bacterial antagonist *Pseudomonas fluorescens* as seed treatment on okra. Indian J. Nematol. 40, 226–228.

[B54] Larriba E. Jaime M. D. L. A. Carbonell-Caballero J. Conesa A. Dopazo J. Nislow C. . (2014). Sequencing and functional analysis of the genome of a nematode egg-parasitic fungus, *Pochonia chlamydosporia*. Fungal Genet. Biol. 65, 69–80. doi: 10.1016/j.fgb.2014.02.002 24530791

[B55] Leal S. C. M. Bertioli D. Ball B. V. Butt T. M. (1994). The presence of double-stranded RNA and virus-like particles in the entomopathogenic fungus *Metarhizium anisopliae*. Biocontrol Sci. Technol. 4, 89–94. doi: 10.1080/0958315940935531637339054

[B56] Li Y. Hyde K. D. Jeewon R. Cai L. Vijaykrishna D. Zhang K. (2005). Phylogenetics and evolution of nematode-trapping fungi (Orbiliales) estimated from nuclear and protein coding genes. Mycologia 97, 1034–1046. doi: 10.3852/mycologia.97.5.1034 16596955

[B57] Lima-Rivera D. Salinas-Castro A. Gutierrez-García G. Rivera-Fernández A. Cerdan C. Villain L. . (2025). Alternatives for the management of coffee corky-root disease: Search for tolerance and antagonistic fungi. Trop. Subtrop. Agroecosystems 28, 083. doi: 10.56369/tsaes.6030

[B58] Lin R. Liu C. Shen B. Bai M. Ling J. Chen G. . (2015). Analysis of the complete mitochondrial genome of *Pochonia chlamydosporia* suggests a close relationship to the invertebrate-pathogenic fungi in Hypocreales. BMC Microbiol. 15, 5. doi: 10.1186/s12866-015-0341-8 25636983 PMC4360972

[B59] Lin R. Qin F. Shen B. Shi Q. Liu C. Zhang X. . (2018). Genome and secretome analysis of *Pochonia chlamydosporia* provide new insight into egg-parasitic mechanisms. Sci. Rep. 8, 1123. doi: 10.1038/s41598-018-19169-5 29348510 PMC5773674

[B60] Llanos-López N. A. Wennrich J. P. Miled J. Ashrafi S. Maier W. Surup F. . (2026). Polydosetins & pullularins—bioactive tetramic acids & cyclodepsipeptides from the endophytic and nematophagous fungus *Polydomus karssenii*. Nat. Prod. Bioprospecting 16, 28. doi: 10.1007/s13659-025-00579-8 41629700 PMC12864567

[B61] López-Bucio J. Pelagio-Flores R. Herrera-Estrella A. (2015). *Trichoderma* as biostimulant: exploiting the multilevel properties of a plant beneficial fungus. Sci. Hortic. 196, 109–123. doi: 10.1016/j.scienta.2015.08.04342574925

[B62] Lopez-Llorca L. V. Gómez-Vidal S. Monfort E. Larriba E. Casado-Vela J. Elortza F. . (2010). Expression of serine proteases in egg-parasitic nematophagous fungi during barley root colonization. Fungal Genet. Biol. 47, 342–351. doi: 10.1016/j.fgb.2010.01.004 20097301

[B63] Lopez-Llorca L. V. Maciá-Vicente J. G. Jansson H. B. (2008). “ Mode of action and interactions of nematophagous fungi,” in Integrated Management and Biocontrol of Vegetable and Grain Crops Nematodes. Eds. Ciancio A. Mukerji K. G. ( Springer, Dordrecht, Netherlands), 51–76. doi: 10.1007/978-1-4020-6063-2_3

[B64] Lopez-Nuñez R. Prieto-Rubio J. Bautista I. Lidón-Cerezuela A. L. Valverde-Urrea M. Lopez-Moya F. . (2025). Chitosan reduces naturally occurring plant pathogenic fungi and increases nematophagous fungus *Purpureocillium* in soil under field conditions. Front. Agron. 6, 1502402. doi: 10.3389/fagro.2024.1502402

[B65] Lopez-Nuñez R. Suarez-Fernandez M. Lopez-Moya F. Lopez-Llorca L. V. (2022). Chitosan and nematophagous fungi for sustainable management of nematode pests. Front. Fungal Biol. 3, 980341. doi: 10.3389/ffunb.2022.980341 37746197 PMC10512356

[B66] Lorito M. Woo S. L. Harman G. E. Monte E. (2010). Translational research on *Trichoderma*: from ’omics to the field. Annu. Rev. Phytopathol. 48, 395–417. doi: 10.1146/annurev-phyto-073009-114314 20455700

[B67] Lozano-Soria A. Picciotti U. Lopez-Moya F. Lopez-Cepero J. Porcelli F. Lopez-Llorca L. V. (2020). Volatile organic compounds from entomopathogenic and nematophagous fungi, repel banana black weevil (*Cosmopolites sordidus*). Insects 11, 509. doi: 10.3390/insects11080509 32781701 PMC7469225

[B68] Lozano-Soria A. Picciotti U. Lopez-Moya F. Lopez-Cepero J. Porcelli F. Lopez-Llorca L. V. (2024). Correction: Lozano-Soria et al. Volatile organic compounds from entomopathogenic and nematophagous fungi, repel banana black weevil (*Cosmopolites sordidus*). Insects 2020, 11, 509. Insects 15, 175. doi: 10.3390/insects15030175 38535408 PMC10970921

[B69] Lozano-Soria A. Piedra-Buena Diaz A. Lopez-Moya F. Valverde-Urrea M. Zubcoff J. J. Martinez-Perez J. E. . (2025). Volatile organic compounds from entomopathogenic and nematophagous fungi repel banana weevil (*Cosmopolites sordidus*) under banana field conditions. PeerJ 13, e19414. doi: 10.7717/peerj.19414 40452941 PMC12126970

[B70] Luambano N. D. Manzanilla-López R. H. Kimenju J. W. Powers S. J. Narla R. D. Wanjohi W. J. . (2015). Effect of temperature, pH, carbon and nitrogen ratios on the parasitic activity of *Pochonia chlamydosporia* on *Meloidogyne incognita*. Biol. Control 80, 23–29. doi: 10.1016/j.biocontrol.2014.09.00342574925

[B71] Luo H. Liu Y. Fang L. Li X. Tang N. Zhang K. (2007). *Coprinus comatus* damages nematode cuticles mechanically with spiny balls and produces potent toxins to immobilize nematodes. Appl. Environ. Microbiol. 73, 3916–3923. doi: 10.1128/AEM.02770-06 17449690 PMC1932715

[B72] Luo H. Mo M. Huang X. Li X. Zhang K. (2004). *Coprinus comatus*: A basidiomycete fungus forms novel spiny structures and infects nematode. Mycologia 96, 1218–1225. doi: 10.1080/15572536.2005.11832870 21148944

[B73] Malmierca M. G. Cardoza R. E. Alexander N. J. McCormick S. P. Hermosa R. Monte E. . (2012). Involvement of *Trichoderma* trichothecenes in the biocontrol activity and induction of plant defense-related genes. Appl. Environ. Microbiol. 78, 4856–4868. doi: 10.1128/AEM.00385-12 22562989 PMC3416386

[B74] Malmierca M. G. McCormick S. P. Cardoza R. E. Alexander N. J. Monte E. Gutiérrez S. (2015). Production of trichodiene by *Trichoderma harzianum* alters the perception of this biocontrol strain by plants and antagonized fungi. Environ. Microbiol. 17, 2628–2646. doi: 10.1111/1462-2920.12506 24813508

[B75] Mankau R. (1962). Soil fungistasis and nematophagous fungi. Phytopathology 52, 611–615.

[B76] Manzanilla-López R. Esteves I. Finetti-Sialer M. M. Hirsch P. R. Ward E. Devonshire J. . (2013). *Pochonia chlamydosporia*: Advances and challenges to improve its performance as a biological control agent of sedentary endo-parasitic nematodes. J. Nematol. 45, 1–7. PMC3625126 23589653

[B77] McCormick S. P. Stanley A. M. Stover N. A. Alexander N. J. (2011). Trichothecenes: From simple to complex mycotoxins. Toxins 3, 802–814. doi: 10.3390/toxins3070802 22069741 PMC3202860

[B78] McLean K. S. Lawrence G. W. (1995). Development of *Heterodera glycines* as affected by *Fusarium solani*, the causal agent of sudden death syndrome of soybean. J. Nematol. 27, 70–77. PMC2619589 19277263

[B79] Meel S. Saharan B. S. (2025). Microbial warfare against nematodes: A review of nematicidal compounds for horticulture, environment, and biotechnology. Microbe 9, 100557. doi: 10.1016/j.microb.2025.10055742574925

[B80] Mendonça Rodrigues J. Zanotti Ávila A. de Souza Gouveia A. de Paula Alves Pinto I. da Silva Bonjour M. Strozi Alves Meira R. M. . (2024). The nematophagous root endophyte *Pochonia chlamydosporia* enhances tolerance to drought in soybean. Theor. Exp. Plant Physiol. 36, 727–746. doi: 10.1007/s40626-024-00341-430311153

[B81] Mercado-Blanco J. Abrantes I. Barra Caracciolo A. Bevivino A. Ciancio A. Grenni P. . (2018). Belowground microbiota and the health of tree crops. Front. Microbiol. 9, 1006. doi: 10.3389/fmicb.2018.01006 29922245 PMC5996133

[B82] Mestre-Tomás J. Esgueva-Vilá D. Fuster-Alonso A. Lopez-Moya F. Lopez-Llorca L. V. (2023). Chitosan modulates volatile organic compound emission from the biocontrol fungus *Pochonia chlamydosporia*. Molecules 28, 4053. doi: 10.3390/molecules28104053 37241794 PMC10223989

[B83] Mincuzzi A. Picciotti U. Sanzani S. M. Garganese F. Palou L. Addante R. . (2023). Postharvest diseases of pomegranate: Alternative control means and a spiderweb effect. J. Fungi 9, 808. doi: 10.3390/jof9080808 37623578 PMC10456121

[B84] Monteiro T. S. A. Oliveira A. P. Xavier A. S. Alfenas-Zerbini P. Freitas L. G. (2016). Viral infection in nematophagous fungus *Pochonia chlamydosporia*. J. Nematol. 48, 354.

[B85] Morrell J. J. Bloom J. R. (1981). Influence of *Meloidogyne incognita* on *Fusarium* wilt of tomato at or below the minimum temperature for wilt development. J. Nematol. 13, 57–60. PMC2618047 19300723

[B86] Morton C. O. Mauchline T. H. Kerry B. R. Hirsch P. R. (2003). PCR-based DNA fingerprinting indicates host-related genetic variation in the nematophagous fungus *Pochonia chlamydosporia*. Mycol. Res. 107, 198–205. doi: 10.1017/s0953756203007251 12747331

[B87] Nahar K. Kyndt T. De Vleesschauwer D. Höfte M. Gheysen G. (2011). The jasmonate pathway is a key player in systemically induced defense against root knot nematodes in rice. Plant Physiol. 157, 305–316. doi: 10.1104/pp.111.177576 21715672 PMC3165880

[B88] Nitao J. K. Meyer S. L. Schmidt W. F. Fettinger J. C. Chitwood D. J. (2001). Nematode-antagonistic trichothecenes from *Fusarium equiseti*. J. Chem. Ecol. 27, 859–871. doi: 10.1023/A:1010390200571 11471940

[B89] Oka Y. Shapira N. Fine P. (2007). Control of root-knot nematodes in organic farming systems by organic amendments and soil solarization. Crop Prot. 26, 1556–1565. doi: 10.1016/j.cropro.2007.01.00342574925

[B90] Okungbowa F. I. Shittu H. O. (2012). *Fusarium* wilts: an overview. Environ. Res. J. 6, 83–101.

[B91] Olatinwo R. Yin B. Becker J. O. Borneman J. (2006). Suppression of the plant-parasitic nematode *Heterodera schachtii* by the fungus *Dactylella oviparasitica*. Phytopathology 96, 111–114. doi: 10.1094/PHYTO-96-0111 18944211

[B92] Olmedo-Monfil V. Casas-Flores S. (2014). “ Molecular mechanisms of biocontrol in *Trichoderma* spp. and their applications in agriculture,” in Biotechnology and Biology of Trichoderma. Eds. Gupta V. K. Schmoll M. Herrera-Estrella A. Upadhyay R. S. Druzhinina I. Tuohy M. G. ( Elsevier, Oxford, UK), 429–453. doi: 10.1016/B978-0-444-59576-8.00032-1

[B93] Oskiera M. Szczech M. Bartoszewski G. (2015). Molecular identification of *Trichoderma* strains collected to develop plant growth-promoting and biocontrol agents. J. Hortic. Res. 23, 75–86. doi: 10.2478/johr-2015-0010

[B94] Ozalvo R. Cabrera J. Escobar C. Christensen S. A. Borrego E. J. Kolomiets M. V. . (2014). Two closely related members of *Arabidopsis* 13-lipoxygenases (13-LOXs), LOX3 and LOX4, reveal distinct functions in response to plant-parasitic nematode infection. Mol. Plant Pathol. 15, 319–332. doi: 10.1111/mpp.12094 24286169 PMC6638665

[B95] Patidar P. Prasad L. Sagar S. Sirohi A. Saharan M. S. Dhillon M. K. . (2024). Chemo-profiling of *Purpureocillium lilacinum* and *Paecilomyces variotii* isolates using GC-MS analysis, and evaluation of their metabolites against *M. incognita*. PloS One 19, e0297925. doi: 10.1371/journal.pone.0297925 38358978 PMC10868743

[B96] Pentimone I. Colagiero M. Ferrara M. Nigro F. Rosso L. C. Ciancio A. (2019). Time-dependent effects of *Pochonia chlamydosporia* endophytism on gene expression profiles of colonized tomato roots. Appl. Microbiol. Biotechnol. 103, 8511–8527. doi: 10.1007/s00253-019-10058-z 31392375

[B97] Pentimone I. Lebrón R. Hackenberg M. Rosso L. C. Colagiero M. Nigro F. . (2018). Identification of tomato miRNAs responsive to root colonization by endophytic *Pochonia chlamydosporia*. Appl. Microbiol. Biotechnol. 102, 907–919. doi: 10.1007/s00253-017-8608-7 29105020

[B98] Pereira C. G. Santana Varejão J. O. Ferreira Barros A. Costa da Cruz L. Rocha Fernandes D. Grassi de Freitas L. . (2026). Volatile organic compounds from nematophagous fungi with nematicidal activity against the root-lesion nematode *Pratylenchus brachyurus*. J. Phytopathol. 174, e70231. doi: 10.1111/jph.7023140046247

[B99] Picciotti U. Araujo Dalbon V. Ciancio A. Colagiero M. Cozzi G. De Bellis L. . (2023). Ectomosphere: Insects and microorganism interactions. Microorganisms 11, 440. doi: 10.3390/microorganisms11020440 36838405 PMC9967823

[B100] Rahman M. U. Chen P. Zhang X. Fan B. (2023). Predacious strategies of nematophagous fungi as bio-control agents. Agronomy 13, 2685. doi: 10.3390/agronomy1311268530654563

[B101] Regaieg H. Ciancio A. Horrigue Raouani N. Rosso L. (2011). Detection and biocontrol potential of *Verticillium leptobactrum* parasitizing *Meloidogyne* spp. World J. Microbiol. Biotechnol. 27, 1615–1623. doi: 10.1007/s11274-010-0615-030311153

[B102] Reiner Driéli A. Dallemole-Giaretta R. dos Santos I. Oldoni T. L. C. Lopes E. A. Chiarani A. (2016). Efeito nematicida de um subproduto da indústria vinícola em *Meloidogyne javanica* (Treub) Chitwood. Cienc. Tec. Vitivinic. 31, 24–30. doi: 10.1051/ctv/20163101024

[B103] Richard J. L. (2007). Some major mycotoxins and their mycotoxicoses – an overview. Int. J. Food Microbiol. 119, 3–10. doi: 10.1016/j.ijfoodmicro.2007.07.019 17719115

[B104] Rincy M. K. Eapen S. J. Praveena R. (2025). Exploring the biocontrol potential of *Pochonia chlamydosporia*: antifungal and nematicidal activities of culture filtrates and secondary metabolites. Chem. Biodivers. 22, e01418. doi: 10.1002/cbdv.202501418 40802922

[B105] Robb J. Lee B. (1986). Ultrastructure of mature and fired gun cells of *Haptoglossa mirabilis*. Can. J. Bot. 64, 1935–1947. doi: 10.1139/b86-25734819996

[B106] Rosso L. C. Colagiero M. Salatino N. Ciancio A. (2014). Observations on the effect of trophic conditions on *Pochonia chlamydosporia* gene expression. Ann. Appl. Biol. 164, 232–243. doi: 10.1111/aab.1209940046247

[B107] Rosso L. C. Finetti-Sialer M. M. Hirsch P. R. Ciancio A. Kerry B. R. Clark I. M. (2011). Transcriptome analysis shows differential gene expression in the saprotrophic to parasitic transition of *Pochonia chlamydosporia*. Appl. Microbiol. Biotechnol. 90, 1981–1994. doi: 10.1007/s00253-011-3282-7 21541788

[B108] Rubner A. (1996). Revision of predacious hyphomycetes in the *Dactylella*-*Monacrosporium* complex. Stud. Mycol. 39, 1–134.

[B109] Sánchez-Gómez T. Harte S. J. Zamora P. Bareyre M. Díez J. J. Herrero B. . (2023). Nematicidal effect of *Beauveria* species and the mycotoxin beauvericin against pinewood nematode *Bursaphelenchus xylophilus*. Front. For. Glob. Change 6, 1229456. doi: 10.3389/ffgc.2023.1229456

[B110] Sánchez-Martínez E. Aguilar-Marcelino L. Hernández-Romano J. Castañeda-Ramírez G. S. Mendoza-de Gives P. (2023). Taxonomic and biological characterization and predatory activity of four nematophagous fungi isolates of *Arthrobotrys* species from Tapachula, Chiapas, Mexico. Arch. Agron. Soil Sci. 69, 327–343. doi: 10.1080/03650340.2021.199414137339054

[B111] Sanzani S. M. Gallone T. Garganese F. Caruso A. G. Amenduni M. Ippolito A. (2019). Contamination of fresh and dried tomato by Alternaria toxins in southern Italy. Food Addit. Contam. Part A 36, 789–799. doi: 10.1080/19440049.2019.1588998 30943118

[B112] Scholler M. Hagedorn G. Rubner A. (1999). A reevaluation of predatory orbiliaceous fungi. II. A new generic concept. Sydowia 51, 89–113.

[B113] Sellitto V. M. Curto G. Dallavalle E. Ciancio A. Colagiero M. Pietrantonio L. . (2016). Effect of *Pochonia chlamydosporia*-based formulates on the regulation of root-knot nematodes and plant growth response. Front. Life. Sci. 9, 177–181. doi: 10.1080/21553769.2016.119382737339054

[B114] Shaliha B. A. Swarnakumari N. Anita B. Thiribhuvanamala G. Suganthi A. Saranya N. (2025). The mechanistic pathways of *Pochonia chlamydosporia*: a biology perspective on nematode suppression and plant promotion. Plant Sci. Today 12, 10330. doi: 10.14719/pst.10330

[B115] Sharon E. Bar-Eyal M. Chet I. Herrera-Estrella A. Kleifeld O. Spiegel Y. (2001). Biological control of the root-knot nematode *Meloidogyne javanica* by *Trichoderma harzianum*. Phytopathology 91, 687–693. doi: 10.1094/PHYTO.2001.91.7.687 18942999

[B116] Sharon E. Chet I. Viterbo A. Bar-Eyal M. Nagan H. Samuels G. J. . (2007). Parasitism of *Trichoderma* on *Meloidogyne javanica* and role of the gelatinous matrix. Eur. J. Plant Pathol. 118, 247–258. doi: 10.1007/s10658-007-9140-x30311153

[B117] Shemshura O. N. Bekmakhanova N. E. Mazunina M. N. Meyer S. L. Rice C. P. Masler E. P. (2016). Isolation and identification of nematode-antagonistic compounds from the fungus *Aspergillus candidus*. FEMS Microbiol. Lett. 363, fnw026. doi: 10.1093/femsle/fnw026 26850440

[B118] Shimada A. Fujioka S. Koshino H. Kimura Y. (2010). Nematicidal activity of beauvericin produced by the fungus *Fusarium bulbicola*. Z. Naturforsch. C. 65, 207–210. doi: 10.1515/znc-2010-3-407 20469639

[B119] Shukuru B. N. Politaeva N. A. Sharma N. R. Akhtar N. Ts A. Rana M. (2024). Bioagents and beyond: harnessing the diversity of nematophagous microorganisms and predators for sustainable management of plant–parasitic nematodes. J. Phytopathol. 172, e70005. doi: 10.1111/jph.7000540046247

[B120] Spiegel Y. Chet I. (1998). Evaluation of *Trichoderma* spp. as a biocontrol agent against soilborne fungi and plant-parasitic nematodes in Israel. Integr. Pest Manage. Rev. 3, 169–175. doi: 10.1023/A:100962583112841886696

[B121] Sreeja P. Reji Rani O. P. Chellappan M. (2025). *Lecanicillium saksenae*: a novel nematophagous fungus against root knot nematode, *Meloidogyne incognita* (Kofoid & White 1919) from Kerala, India. J. Asia-Pac. Entomol. 28, 102416. doi: 10.1016/j.aspen.2025.10241642574925

[B122] Stucky T. Sy E. T. Egger J. Mathlouthi E. Krauss J. De Gianni L. . (2024). Control of the plant-parasitic nematode *Meloidogyne incognita* in soil and on tomato roots by *Clonostachys rosea*. J. Appl. Microbiol. 135, lxae111. doi: 10.1093/jambio/lxae111 38692851

[B123] Sun Z. B. Li S. D. Ren Q. Xu J. L. Lu X. Sun M. H. (2020). Biology and applications of *Clonostachys rosea*. J. Appl. Microbiol. 129, 486–495. doi: 10.1111/jam.14625 32115828

[B124] Sung G. H. Hywel-Jones N. L. Sung J. M. Luangsa-ard J. J. Shrestha B. Spatafora J. W. (2007a). Phylogenetic classification of *Cordyceps* and the clavicipitaceous fungi. Stud. Mycol. 57, 5–59. doi: 10.3114/sim.2007.57.01 18490993 PMC2104736

[B125] Sung G. H. Sung J. M. Hywel-Jones N. L. Spatafora J. W. (2007b). A multi-gene phylogeny of Clavicipitaceae (Ascomycota, Fungi): identification of localized incongruence using a combinational bootstrap approach. Mol. Phylogenet. Evol. 44, 1204–1223. doi: 10.1016/j.ympev.2007.03.011 17555990

[B126] Swarnakumari N. Sindhu R. Thiribhuvanamala G. Rajaswaminathan V. (2020). Evaluation of oil dispersion formulation of nematophagus fungus, *Pochonia chlamydosporia* against root-knot nematode, *Meloidogyne incognita* in cucumber. J. Asia-Pac. Entomol. 23, 1283–1287. doi: 10.1016/j.aspen.2020.10.00842574925

[B127] Swe A. Jeewon R. Pointing S. B. Hyde K. D. (2009). Diversity and abundance of nematode-trapping fungi from decaying litter in terrestrial, freshwater and mangrove habitats. Biodivers. Conserv. 18, 1695–1714. doi: 10.1007/s10531-008-9553-730311153

[B128] Tarigan W. E. Munir E. Hastuti L. D. S. Hartanto A. (2020). Occurrence of nematophagous fungi in freshwater samples of Toba Lake, North Sumatra, Indonesia. J. Phys. Conf. Ser. 1462, 12052. doi: 10.1088/1742-6596/1462/1/012052

[B129] Tijerino A. Cardoza R. E. Moraga J. Malmierca M. G. Vicente F. Aleu J. . (2011). Overexpression of the trichodiene synthase gene tri5 increases trichodermin production and antimicrobial activity in *Trichoderma brevicompactum*. Fungal Genet. Biol. 48, 285–296. doi: 10.1016/j.fgb.2010.11.012 21145409

[B130] Tolba S. R. T. Rosso L. C. Pentimone I. Colagiero M. Moustafa M. M. A. Elshawaf I. I. S. . (2021). Root endophytism by *Pochonia chlamydosporia* affects defense-gene expression in leaves of monocot and dicot hosts under multiple biotic interactions. Plants 10, 718. doi: 10.3390/plants10040718 33917204 PMC8068004

[B131] Toledo da Silva A. Castro de Souza D. Amorim de Souza S. de Souza Alves J. C. Souza Dias E. Aguilar-Marcelino L. . (2024). Linking the protease activity to the nematicidal action of edible mushroom. World J. Microbiol. Biotechnol. 40, 170. doi: 10.1007/s11274-024-03980-6 38630319

[B132] Uthoff J. Jakobs-Schönwandt D. Dietz K. J. Patel A. (2024a). Development of a seed treatment with *Pochonia chlamydosporia* for biocontrol application. Agriculture 14, 138. doi: 10.3390/agriculture1401013830654563

[B133] Uthoff J. Jakobs-Schönwandt D. Schmidt J. H. Hallmann J. Dietz K. J. Patel A. (2024b). Biological enhancement of the cover crop *Phacelia tanacetifolia* (Boraginaceae) with the nematophagous fungus *Pochonia chlamydosporia* to control the root-knot nematode *Meloidogyne hapla* in a succeeding tomato plant. BioControl 69, 77–90. doi: 10.1007/s10526-023-10222-530311153

[B134] Verdejo-Lucas S. Ornat C. Sorribas F. J. Stchiegel A. (2002). Species of root-knot nematodes and fungal egg parasites recovered from vegetables in Almería and Barcelona, Spain. J. Nematol. 34, 405–408. PMC2620581 19265964

[B135] Viaene N. M. Abawi G. S. (1998). Fungi parasitic on juveniles and egg masses of *Meloidogyne hapla* in organic soils from New York. J. Nematol. 30, 632–638. PMC2620331 19274258

[B136] Villarreal-Pérez C. J. Collantes-González R. D. Pitti-Caballero J. Peraza-Padilla W. Hofmann T. A. (2025). Nematophagous fungi for integrated management of *Meloidogyne* (Tylenchida): a review of taxonomic diversity, mechanisms of action and potential as biological control agents. Sci. Agropecu. 16, 541–556. doi: 10.17268/sci.agropecu.2025.041

[B137] Vinale F. Marra R. Scala F. Ghisalberti E. L. Lorito M. Sivasithamparam K. (2006). Major secondary metabolites produced by two commercial *Trichoderma* strains active against different phytopathogens. Lett. Appl. Microbiol. 43, 143–148. doi: 10.1111/j.1472-765X.2006.01939.x 16869896

[B138] Vinale F. Sivasithamparam K. Ghisalberti E. L. Ruocco M. Wood S. L. Lorito M. (2012). *Trichoderma* secondary metabolites that affect plant metabolism. Nat. Prod. Commun. 7, 1545–1550. doi: 10.1177/1934578X120070113 23285827

[B139] Vizcaíno J. A. Sanz L. Basilio A. Vicente F. Gutiérrez S. Hermosa M. R. . (2005). Screening of antimicrobial activities in *Trichoderma* isolates representing three trichoderma sections. Mycol. Res. 109, 1397–1406. doi: 10.1017/s0953756205003898 16353639

[B140] Vos C. Geerinckx K. Mkandawire R. Panis B. De Waele D. Elsen A. (2012). Arbuscular mycorrhizal fungi affect both penetration and further life stage development of root-knot nematodes in tomato. Mycorrhiza 22, 157–163. doi: 10.1007/s00572-011-0422-y 22147206

[B141] Wallingford A. K. Cha D. H. Linn C. E. Jr Wolfin M. S. Loeb G. M. (2017). Robust manipulations of pest insect behavior using repellents and practical application for integrated pest management. Environ. Entomol. 46, 1041–1050. doi: 10.1093/ee/nvx125 28981656

[B142] Wang D. Li Y. Yuan Y. Chu D. Cao J. Sun G. . (2022). Identification of non-volatile and volatile organic compounds produced by *Bacillus siamensis* LZ88 and their antifungal activity against *Alternaria alternata*. Biol. Control 169, 104901. doi: 10.1016/j.biocontrol.2022.10490142574925

[B143] Wang D. Ma N. Rao W. Zhang Y. (2023). Recent advances in life history transition with nematode-trapping fungus *Arthrobotrys oligospora* and its application in sustainable agriculture. Pathogens 12, 367. doi: 10.3390/pathogens12030367 36986289 PMC10056792

[B144] Ward E. Kerry B. R. Manzanilla-López R. H. Mutua G. Devonshire J. Kimenju J. . (2012). The *Pochonia chlamydosporia* serine protease gene vcp1 is subject to regulation by carbon, nitrogen and pH: implications for nematode biocontrol. PloS One 7, e35657. doi: 10.1371/journal.pone.0035657 22558192 PMC3338732

[B145] Westphal A. Becker J. O. (2001). Components of soil suppressiveness against *Heterodera schachtii*. Soil Biol. Biochem. 33, 9–16. doi: 10.1016/S0038-0717(00)00108-5

[B146] Witte H. Yang J. Logan G. D. Colindres N. B. Peacock B. B. Becker J. S. . (2020). *Hyalorbilia oviparasitica* clade detected in field soils cropped to sugar beets and enriched in the presence of *Heterodera schachtii* and a host crop. PhytoFrontiers 1, 13–20. doi: 10.1094/PHYTOFR-07-20-0005-R

[B147] Wolstrup J. Nansen P. Grønvold J. Henriksen S. A. Larsen M. (1996). Toward practical biological control of parasitic nematodes in domestic animals. J. Nematol. 28, 129–132. PMC2619686 19277128

[B148] Yang Y. Liu X. Z. (2006). A new generic approach to the taxonomy of predatory anamorphic Orbiliaceae (Ascomycotina). Mycotaxon 97, 153–161. doi: 10.5962/p.419131

[B149] Yang Y. Yang E. An Z. Liu X. (2007). Evolution of nematode-trapping cells of predatory fungi of the Orbiliaceae based on evidence from rRNA-encoding DNA and multiprotein sequences. Proc. Natl. Acad. Sci. U.S.A. 104, 8379–8384. doi: 10.1073/pnas.0702770104 17494736 PMC1895958

[B150] Zare R. Gams W. (2001). A revision of *Verticillium* section *Prostrata*. VI. The genus *Haptocillium*. Nova Hedwig. 73, 271–292.

[B151] Zavala-Gonzalez E. A. Rodríguez-Cazorla E. Escudero N. Aranda-Martinez A. Martínez-Laborda A. Ramírez-Lepe M. . (2017). *Arabidopsis thaliana* root colonization by the nematophagous fungus *Pochonia chlamydosporia* is modulated by jasmonate signaling and leads to accelerated flowering and improved yield. New Phytol. 213, 351–364. doi: 10.1111/nph.14106 27456071

[B152] Zhang S. Gan Y. Ji W. Xu B. Hou B. Liu J. (2017). Mechanisms and characterization of *Trichoderma longibrachiatum* T6 in suppressing nematodes (*Heterodera avenae*) in wheat. Front. Plant Sci. 8, 1491. doi: 10.3389/fpls.2017.01491 28966623 PMC5605630

[B153] Zhang L. Zhang T. Xu Y. R. Sun J. M. Pan X. R. Gu K. Z. . (2025). Induction of conidial traps in the nematode-trapping fungus *Drechslerella dactyloides* by soil microbes. mSystems 10, e0129124. doi: 10.1128/msystems.01291-24 39945538 PMC11915823

[B154] Zheng H. Chen T. Li W. Hong J. Xu J. Yu Z. (2024). Endosymbiotic bacteria within the nematode-trapping fungus *Arthrobotrys musiformis* and their potential roles in nitrogen cycling. Front. Microbiol. 15, 1349447. doi: 10.3389/fmicb.2024.1349447 38348183 PMC10860758

[B155] Zoubi B. Mokrini F. Lahlali R. Ghoulam C. Hosseini A. I. I. Qaddoury A. (2025). Evaluating the efficacy of *Aspergillus* bio-agents against the citrus nematode *Tylenchulus semipenetrans*: Laboratory and greenhouse trials. Physiol. Mol. Plant Pathol. 136, 102540. doi: 10.1016/j.pmpp.2024.10254042574925

